# Charge-Dependent Crossover in Aqueous Organic Redox Flow Batteries Revealed Using Online NMR Spectroscopy

**DOI:** 10.1021/acs.jpclett.3c03482

**Published:** 2024-02-01

**Authors:** Emma J. Latchem, Thomas Kress, Peter A. A. Klusener, R. Vasant Kumar, Alexander C. Forse

**Affiliations:** † Yusuf Hamied Department of Chemistry, University of Cambridge, Lensfield Rd., Cambridge CB2 1EW, U.K.; ‡ Department of Materials Science, University of Cambridge, Charles Babbage Rd., Cambridge CB3 0FS, U.K.; § Shell Global Solutions International B.V., Energy Transition Campus, Grasweg 31, Amsterdam 1031 HW, Netherlands

## Abstract

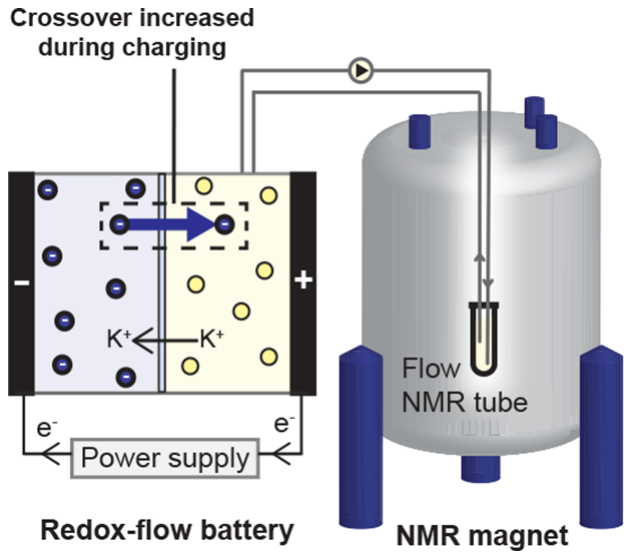

Aqueous organic redox-flow batteries (AORFBs) are promising candidates for low-cost grid-level energy storage. However, their wide-scale deployment is limited by crossover of redox-active material through the separator membrane, which causes capacity decay. Traditional membrane permeability measurements do not capture all contributions to crossover in working batteries, including migration and changes in ion size and charge. Here we present a new method for characterizing crossover in operating AORFBs using online ^1^H NMR spectroscopy. By the introduction of a separate pump to decouple NMR and battery flow rates, this method opens a route to quantitative time-resolved monitoring of redox-flow batteries under real operating conditions. In this proof-of-concept study of a 2,6-dihydroxyanthraquinone (2,6-DHAQ)/ferrocyanide model system, we observed a doubling of the 2,6-DHAQ crossover during battery charging, which we attribute to migration effects. This new membrane testing methodology will advance our understanding of crossover and accelerate the development of improved redox-flow batteries.

Tackling the climate crisis requires huge increases in renewable power generation from the wind and sun.^1^ Established technologies are too expensive to regulate their intermittent energy output on a large scale;^2,3^ hence, research into aqueous organic redox-flow batteries (AORFBs) has recently intensified.^4−7^ An AORFB consists of two aqueous electrolytes (anolyte and catholyte) separated by an ion-selective membrane (Figure 1). The electrolytes are pumped into the electrochemical cell, where they are charged or discharged. Utilizing a modular design and organic redox-active materials, these batteries have the potential to meet cost, scalability, safety, and sustainability targets.^6−9^ However, the lifetime of these batteries is limited by crossover-driven capacity fade.^8−10^

**Figure 1:**
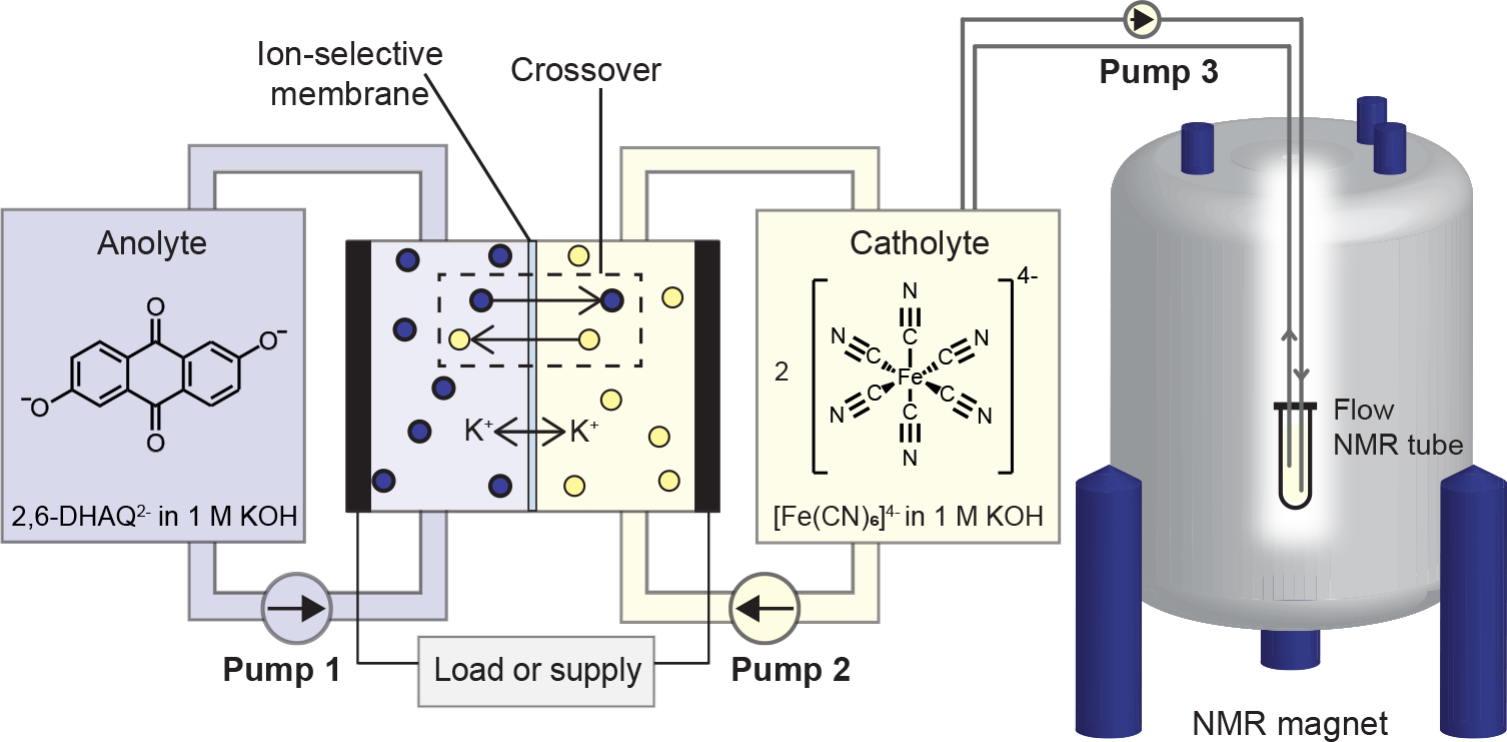
Online NMR setup for measuring crossover in the 2,6-DHAQ/ferrocyanide redox-flow battery system with online NMR analysis. Crossover through the ion-selective membrane is schematically depicted for 2,6-DHAQ^2–^ molecules (represented as blue spheres) and catholyte [Fe(CN)_6_]^4–^ (represented as yellow spheres).

“Crossover” describes the unwanted transport of redox-active components through the membrane. This is generally assessed using single-electrolyte diffusion-cell experiments,^9−14^ though the measured permeabilities do not always correlate well with battery performance.^9^ In operating batteries, the oxidation states (and hence charge density) of redox-active species change and there are additional transport mechanisms to consider when a charge is applied to a cell (e.g., migration and electro-osmosis).^15,16^ Both the charge and size of the redox-active species have been shown to be important determiners of permeability rates through Nafion ion-exchange membranes, with charge being the dominant factor.^14^ However, these effects have not yet been studied in the battery environment, where there will likely be a trade-off between increased membrane repulsion and increased migration as a result of a higher effective charge. To further advance membrane design, it is important that all the contributions to crossover in operating AORFBs are fully understood and measured.^10,11,15^ Though migration is a well-known phenomenon, its contribution to crossover has only been estimated based on electrochemical and theoretical studies.^13,15−17^ Here we report the first direct *in situ* measurements of the crossover in a fully operating AORFB.

Here, to investigate the impact of battery operation on crossover, we develop a new three-pump configuration for online solution-state ^1^H NMR spectroscopy (Figure 1). In previous *in situ* spectroscopic redox-flow battery studies,^18−22^ a two-pump configuration was used, meaning that the flow rate supplying the NMR and redox-flow battery were kept the same. The flow rate needed for quantitative NMR is an order of magnitude lower than the flow rate needed for a functional redox-flow battery. The flow rate affects both battery electrochemistry and crossover rates, so it is important to study the battery under real operating conditions.^11^ Here, a third pump is therefore used to decouple the NMR and AORFB flow, enabling quantitative ^1^H NMR detection without compromising the battery operating conditions (see Methods S1–S9 and Figures S1–S2 in the Supporting Information).

For this study we focus on the 2,6-dihydroxyanthraquinone (2,6-DHAQ)/ferrocyanide battery^7^ (Scheme 1 and Figure 1) equipped with a Nafion membrane, as it is often used as a benchmark model system.^18,19,21,23,24^ We find that battery operation has a significant impact on the 2,6-DHAQ crossover. In particular, we observe that the 2,6-DHAQ crossover rate increases with the current applied during constant-current charging steps, revealing that battery operation is an important factor to consider in any crossover-mitigation strategies.

**Scheme 1:**
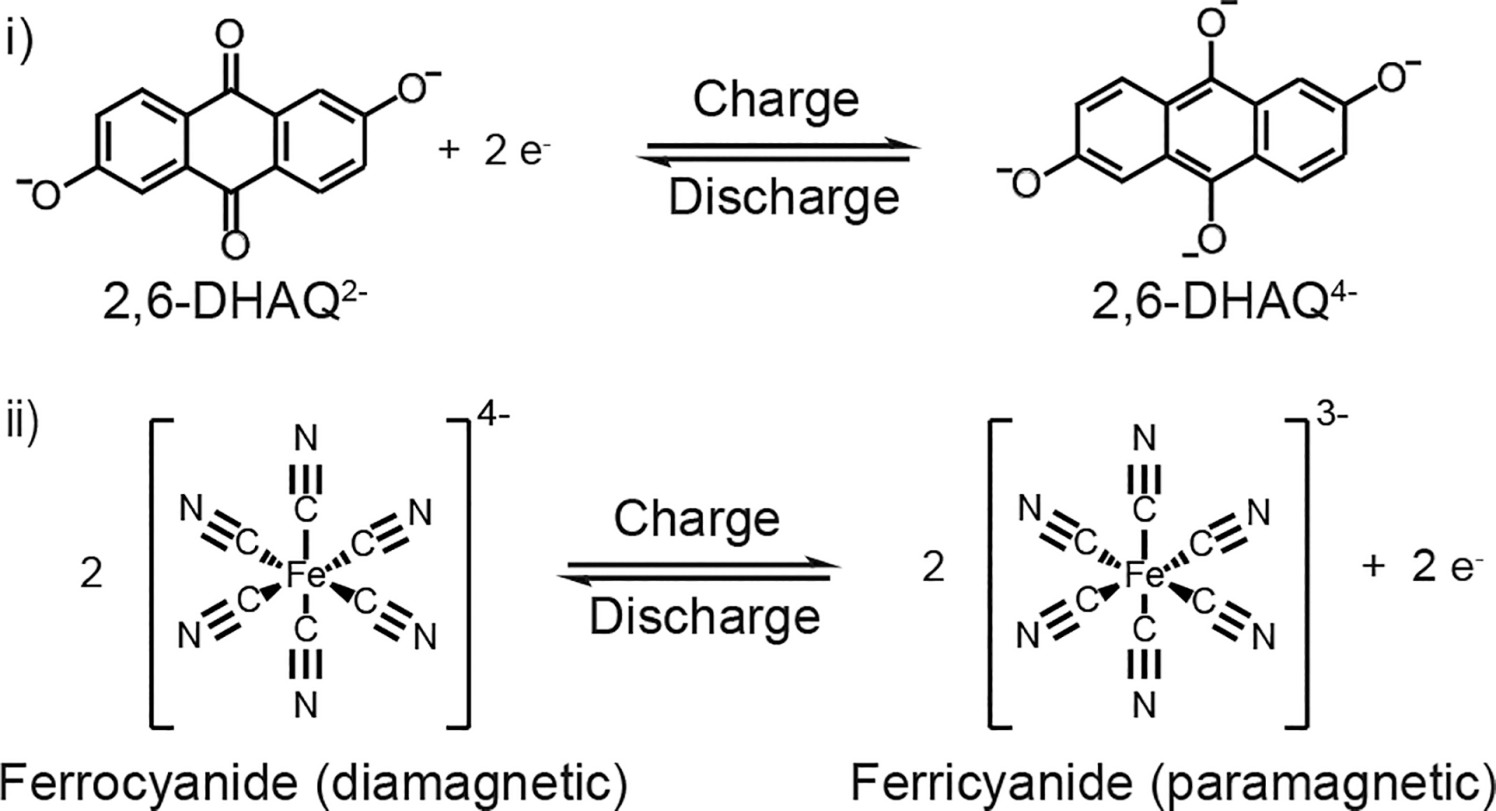
Half-Reactions for the (i) Anolyte and (ii) Catholyte

The suitability of our online NMR method was first demonstrated by measuring the background crossover of 2,6-DHAQ in a redox-flow battery without any applied voltage. Throughout the experiment, ^1^H NMR spectra of the catholyte are continuously collected via a flow NMR tube; each spectrum collected represents an average of the system over 5 min. Sample flow can lead to signal suppression at higher flow rates,^25^ so care was taken to ensure that all the NMR spectra were quantitative for 2,6-DHAQ (see Tables S1–S4, Figures S3–S4 and Methods S4–S6 in the Supporting Information). The crossover of 2,6-DHAQ was detected by the appearance of the three characteristic aromatic proton resonances in the catholyte spectra, which increase in intensity over time (Figure 2a). Interestingly, the chemical shifts of these peaks also decrease over time, which can be explained by changes in intermolecular interactions between 2,6-DHAQ^2–^ ions at higher concentrations^26^ and changes in bulk magnetism, due to ferricyanide crossover.^22,27^ By quantifying the signal intensity for 2,6-DHAQ^2–^ proton A (H_A_), we observed that the concentration of 2,6-DHAQ in the catholyte linearly increases over the duration of the experiment (Figure 2b, cell 1). The rate of increase in 2,6-DHAQ concentration was used to estimate a permeability (see Methods S9 and Equation S1). Our measured 2,6-DHAQ permeability for cell 1, (4.41 ± 0.01) × 10^–10^ cm^2^ s^–1^, is within the range reported in the literature.^9,12^ However, we note that the previously reported permeability values vary significantly, highlighting another challenge with using this metric to assist with membrane testing and design. Our findings demonstrate that online NMR spectroscopy can be used as an alternative *in situ* method for measuring real background permeabilities in redox-flow batteries.

**Figure 2:**
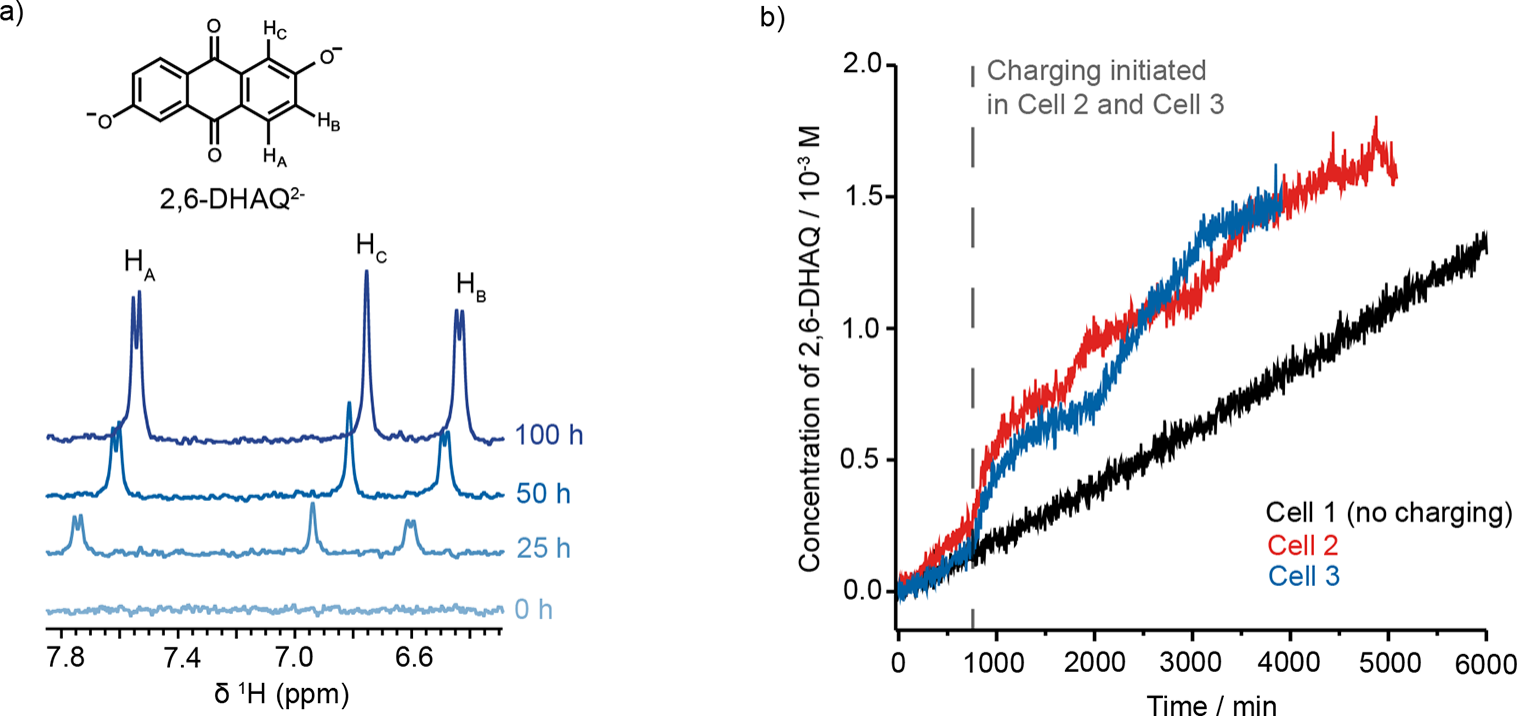
Crossover measurements in redox-flow batteries. (a) Selected ^1^H NMR spectra (9.4 T) from the catholyte side of a resting AORFB cell (cell 1), where there is electrolyte flow but no charging. (b) Plot showing the concentration of 2,6-DHAQ in the catholyte side during crossover experiments performed on a resting cell (cell 1) and two operating cells (cells 2 and 3) cycled using the protocols detailed in Figures S5–S6 and Methods S2. The dashed line indicates the point in time at which charging was initiated in cells 2 and 3.

Encouraged by these initial results, we then applied this technique to an operating battery (Figure 2b). Two identical cells (cells 2 and 3) were prepared and cycled between 0.6 and 1.5 V at three different currents (50, 25, and 10 mA), with rest periods in between. To identify any temporal effects, the order in which each current was applied was varied between cells 2 and 3 (see Figures S5 and S6). We note that both reduced and oxidized 2,6-DHAQ are now present in the anolyte side; nevertheless, due to the relative redox potentials, 2,6-DHAQ is only present in its oxidized form when it is detected by NMR on the catholyte side (see Figures S7–S8 and Methods S6). Initially, the 2,6-DHAQ buildup profile looks similar for each cell (Figure 2b); however, once battery charge is initiated after a 12 h rest period, a marked increase in crossover rate is observed in the operating cells (cells 2 and 3). The crossover rates in cells 2 and 3 are no longer constant and now vary depending on the charging protocol used for each cell, demonstrating that the electrochemistry of the system plays an important role in 2,6-DHAQ crossover.

Having observed operation-dependent 2,6-DHAQ crossover rates, we then examined how this related to each step of the battery charging protocol. In Figure 3a,b, the amount of 2,6-DHAQ in the catholyte is plotted along with the state-of-charge of the cell (i.e., the level of charge of the battery relative to its capacity). Accurately determining the state-of-charge of a redox-flow battery is an ongoing challenge in the field. Here we have measured this from the change in water (HOD) ^1^H chemical shift, which changes as a function of ferricyanide concentration and therefore state-of-charge (see Figure S9 and Methods S7) and has previously been shown to be a reliable method.^22^ Using this approach, we found that both cell 2 and cell 3 reached >92% of their theoretical capacity (116 mA h) in the first 50 mA charge, highlighting the good performance of the battery.

**Figure 3:**
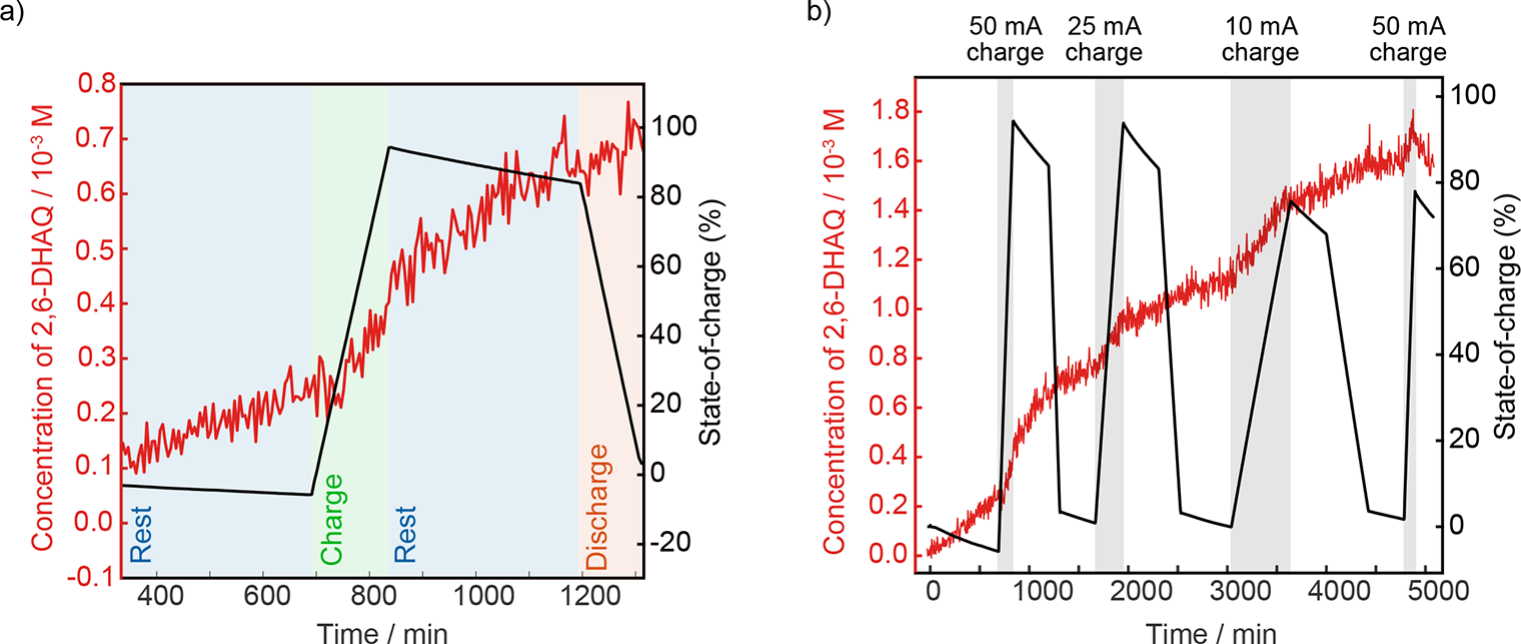
Correlating the 2,6-DHAQ crossover with battery charging. (a) Concentration of 2,6-DHAQ in the operating 2,6-DHAQ/ferrocyanide cell during the initial rest and first charge-rest-discharge cycle (cell 2) correlated to the battery state-of-charge and annotated with the charging mode. (b) Full 2,6-DHAQ crossover data set for cell 2.

Importantly, the periods of enhanced 2,6-DHAQ crossover correspond to the charging steps where the state-of-charge is increasing (Figure 3a,b). These increases in crossover rate are most likely explained by additional transport driven by 2,6-DHAQ migration: During charging, electrons are supplied to the anolyte side of the cell, and in return, K^+^ ions migrate into the anolyte side to balance the charge. However, the charge balance can also be achieved by the movement of negative ions into the catholyte side. In the 2,6-DHAQ/ferrocyanide battery, such a mechanism would manifest as the 2,6-DHAQ crossover rates being positively correlated to charging current. Previous electrochemical and theoretical studies estimate that crossover is increased by this effect, though this has not yet been measured directly, and the contribution to crossover was predicted to be small at currents below 100 mA cm^–2^.^13,16^

To explore the relationship between charging current and 2,6-DHAQ crossover, the crossover rate was measured (moles of 2,6-DHAQ per area of membrane per hour)^9^ for each charging current used (Figure 4a). Note that as we now have a polarized system, Fick’s law of diffusion is no longer appropriate, and crossover rates are therefore used instead of permeabilities (see Methods S9 and Equation S2). This analysis revealed that the increases in 2,6-DHAQ crossover rate during constant-current charge were exacerbated at higher currents (Figure 4a), supporting the conclusion that this effect is driven by the migration of 2,6-DHAQ ions. Though we note that there is some cell-to-cell variability, the magnitude of this effect is significant relative to the calculated errors (Methods S9). Importantly, unlike the predictions from previous theoretical studies,^13,16^ the increase in crossover is significant; migration appears to double the rate of crossover during the 50 mA constant-current charge. This shows that battery operation has a non-negligible impact on crossover even at low currents. Optimization of the charging protocols could also be considered as a route to mitigating crossover.

**Figure 4:**
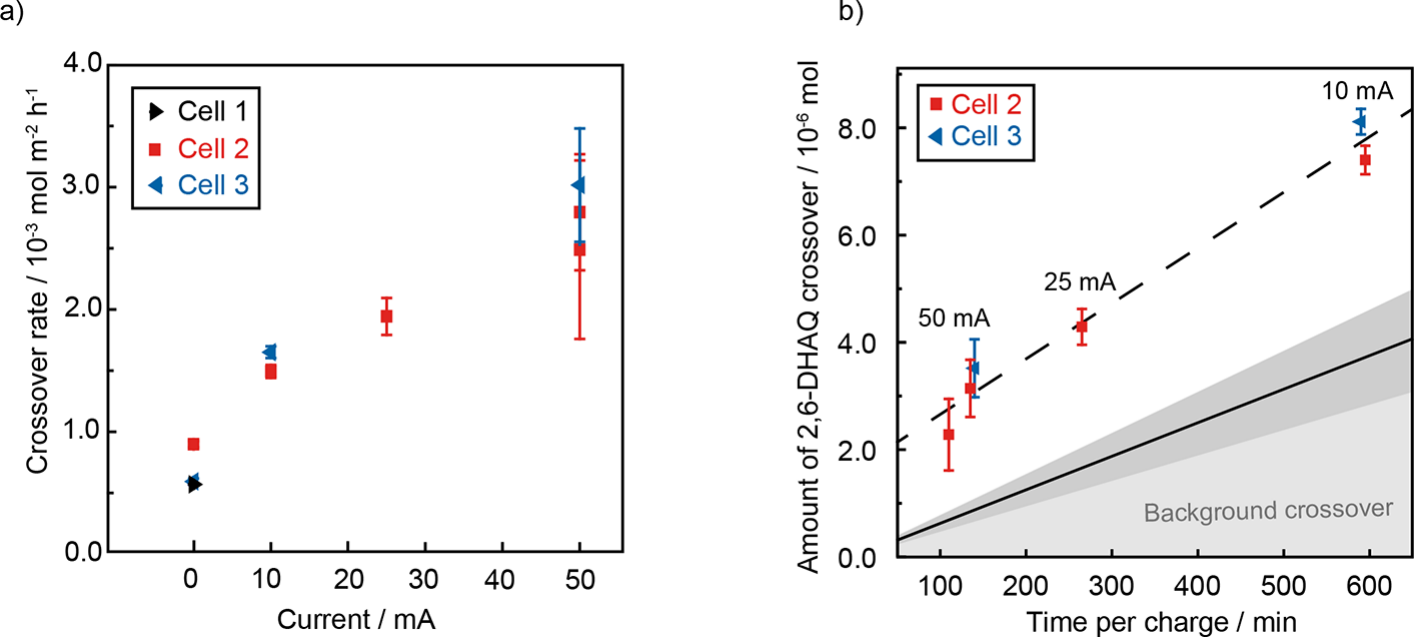
Correlating 2,6-DHAQ crossover with battery charging metrics. (a) 2,6-DHAQ crossover rates correlated to charging current. (b) Total amount of 2,6-DHAQ crossover per constant-current charge, plotted as a function of time per charge and annotated with the charging current used. The gray shaded area shows the calculated contribution from background crossover over time, where the black solid line represents the average between cells 2 and 3, and the dark gray shaded area represents the variation in background crossover between cells (see Methods S9).

When choosing the optimal charging current, there is a trade-off to consider between the crossover rate and the time period per charge. The time taken for a 10 mA charge is approximately five times longer than that for a 50 mA charge. Therefore, although crossover rates are lowest when a low current is used, the time period over which crossover rates are elevated is longer. To determine which charging current is optimal for mitigating crossover, the total amount of 2,6-DHAQ crossover was calculated for each charging period (Figure 4b and Methods S9). Here the background (i.e., diffusional) crossover is assumed to be constant and is estimated from the average background crossover measured at the start of the experiment before battery charging. When the total amount to crossover is compared to the background crossover during one charge period, it appears that the crossover enhancement increases with a decreasing current. Therefore, over the current range examined, the highest current was found to be optimal for mitigating the 2,6-DHAQ crossover during a constant-current charge.

The same crossover rate analysis was also performed for the periods of cell rest and constant-current discharge. Surprisingly, the crossover rates during the rest periods at a higher state-of-charge were slightly elevated compared to those measured at a lower state-of-charge (see Figure S10). As Nafion is a negatively charged cation-exchange membrane, it was expected that there would be more charge repulsion (i.e., Donnan exclusion)^14^ and hence lower crossover for 2,6-DHAQ^4–^ compared to 2,6-DHAQ^2–^. Our results do not support this assumption, possibly indicating that the effective size and/or charge of 2,6-DHAQ^4–^ is smaller than that of 2,6-DHAQ^2–^. This hypothesis could be tested further by performing diffusion-ordered NMR spectroscopy (DOSY) and electrophoretic NMR spectroscopy to the anolyte side of the battery when held at the same state-of-charge.^28,29^ Interestingly, however, this trend is lost toward the end of the experiment for cell 2. We believe that this may be the result of quinone–ferricyanide interactions, which lead to 2,6-DHAQ precipitation from the catholyte at a high state-of-charge. Further studies of these processes are beyond the scope of this work and are the subject of an ongoing study in our lab.

When the crossover rates at rest are compared to those during constant-current discharge of the battery, no significant difference is observed (Figure S11). Migration is expected to operate in the opposite direction during discharge as the polarity of the cell is reversed. However, owing to the significantly lower concentration of 2,6-DHAQ in the catholyte ([2,6-DHAQ] < 2 mM), this effect is overshadowed by the diffusional flux of 2,6-DHAQ into the catholyte from the anolyte side ([2,6-DHAQ] ≈ 100 mM). We therefore conclude that for this system the constant-current charging step is the most influential on 2,6-DHAQ crossover.

Concluding, here we have developed a new online NMR spectroscopy approach for studying electrolyte crossover in operating redox-flow batteries. We found that 2,6-DHAQ crossover rates through Nafion depended on the charging current and that they are doubled during a 50 mA constant-current charge compared to when the cell is at rest. This demonstrates that the transport mechanisms governing crossover vary throughout battery charging cycles and are not adequately captured by simple diffusional permeability studies that do not take electrochemistry into account. Considering that the charging currents used in industry (∼100 mA cm^–2^) are over an order of magnitude higher than those used here (2–10 mA cm^–2^), the impact of this additional transport on the total crossover rate in commercial redox-flow batteries is likely large. Though we have focused on anolyte crossover here, this method is equally applicable to studying the crossover of novel organic catholytes. Importantly, our experiments were all performed using a commercially available flow NMR tube and widespread standard solution-state NMR equipment. No longer bound by the equipment requirements of previous *in situ* NMR configurations,^18,19,21^ this form of rigorous RFB analysis could be performed routinely in most laboratories. Determining key parameters for each contribution to crossover transport will facilitate the development of better transport models and accelerate membrane design.

## Data Availability

All raw experimental data files and supporting code are available in the Cambridge Research Repository, Apollo, with the identifier: 10.17863/CAM.96373.

## Supporting Information Available

Detailed description of experimental methods and materials, including photographs and diagrams of experimental setup (Methods S1–S9, Tables S1–S4, Equations S1–S2, and Figures S1–S19) (PDF)
Transparent Peer Review report available (PDF)
