# Supplementary material for: Charge-Dependent Crossover in Aqueous Organic Redox Flow Batteries Revealed Using Online NMR Spectroscopy
Source: J Phys Chem Lett. 2024 Feb 1;15(5):1515–20. doi: 10.1021/acs.jpclett.3c03482 (PMC10860123; doi:10.1021/acs.jpclett.3c03482)
Supplement: Supplementary file 1 — jz3c03482_si_001.pdf [file jz3c03482_si_001.pdf]

Supporting Information for:

Charge Dependent-Crossover in Aqueous Organic Redox Flow Batteries Revealed Using On-Line NMR Spectroscopy

Emma J. Latchem,[a,b] Thomas Kress,[a] Peter A. A. Klusener,[c] R. Vasant Kumar,[b] Alexander C. Forse[a*]

[a]Yusuf Hamied Department of Chemistry, Lensfield Rd, University of Cambridge, CB2 1EW, UK

[b]Department of Materials Science, University of Cambridge, Charles Babbage Rd, CB3 0FS, UK

[c]Shell Global Solutions International B.V., Energy Transition Campus Amsterdam, Grasweg 31, 1031 HW, NL

*acf50@cam.ac.uk

Contents

Methods S1 4
Methods S2 4
Methods S3 5
Methods S4 5
Methods S5 6
Methods S6 7
Methods S7 7
Methods S8 8
Methods S9 9
Table S1 10
Table S2 10
Table S3 10
Table S4 11
Equation S1 12
Equation S2 12
Figure S1 13
Figure S2 14
Figure S3 15
Figure S4 16
Figure S5 17
Figure S6 17
Figure S7 18
Figure S8 19
Figure S9 20
Figure S10 21
Figure S11 21
Figure S12 22
Figure S13 23
Figure S14 24

Figure S15..25
Figure S16..26
Figure S17..26
Figure S18..27
Figure S19..27
Supplementary references 28

Methods

Methods S1: Materials

The organic redox-active material used in the anolyte was 2,6-dihydroxyanthraquinone (DHAQ, >98% purity, AK Scientific). The inorganic redox-active components used in the catholyte were potassium ferrocyanide trihydrate and potassium ferricyanide (both 99%+ purity, ACROS Organics). All electrolytes were prepared in deuterium oxide (D_2O, 99.98% atom% D, Sigma-Aldrich), with 1 M potassium hydroxide as the supporting electrolyte (KOH, 80% purity, Fisher Chemical). For each experiment, 22 mL anolyte (0.1 M DHAQ) and 22 mL catholyte (0.25 M potassium ferrocyanide trihydrate with 0.04 M potassium ferricyanide) were used. The ion-selective membrane used was Nafion 211 (25 µm dry thickness, FuelCellStore). The Nafion membranes were pre-treated, using the method described by Zhao *et al.*[1] Firstly, they were soaked in MilliQ water at 80 ^{o}C for 20 min. Following this, they were placed in 5 wt% H_2O_2 solution (prepared from 30 wt% H_2O_2 in H_2O, Sigma-Aldrich) for 35 min. Finally, the membranes were rinsed in MilliQ water and then stored in 0.1 M KOH solution for at least 24 h before use. Untreated carbon felt was used as the electrode material (SIGRACELL® battery felt, GFD4,6 EA, SGL Carbon).

Methods S2: Redox Flow Battery apparatus

The redox-flow battery cell end plates, current collectors, flow fields, gaskets and flow fames were all part of a commercial test fixture (Redox Flow Cell Test Fixture, Scribner Associates Inc.) purchased from Alvatek Ltd., Romsey. The redox-flow battery cell used consisted of two aluminium end plates, two gold-plated copper current collectors, two 5 cm^2 graphite serpentine flow fields, four Viton gaskets (0.7 mm thick) and two PTFE flow frames (5 cm^2, 0.080” thick). The flow cell was assembled so that gaskets were placed between the flow fields, PTFE flow frames and a Nafion 211 membrane. One carbon felt electrode (5 cm^2, 4.6 mm thick) was placed in each PTFE flow frame, and the flow cell was sealed with bolts, tightened to 2 N m using a torque wrench. The perfluoroalkoxy polymer inlet and outlet tubes (PFA, 1/8” OD, Swagelok) were inserted into the back of the flow fields, through the aluminium endplates and current collectors. Viton O-ring were used to prevent electrolyte leaks where the inlet and outlet tubing connected to the graphite flow fields.

The electrolyte reservoirs used were custom-made from 50 mL burettes (Pyrex™ Labware Class AS Borosilicate Glass Burette, Pyrex 3295/16AS) with a septum at the top, and a 1/4” OD inlet and outlet near the bottom and top of the reservoir, respectively. A Young’s tap was fitted to the reservoir outlet so that it could be sealed when necessary. The electrolyte reservoirs were designed so that the electrolyte levels could be monitored via a camera (Logitech C920S HD Pro Webcam). Electrolytes were pumped through the flow battery at 60 rpm (~16 mL min^{-1}) using peristaltic pumps (MasterFlex L/S Pump, 07528-10; Easy-load Pump Head, 77202-60; MasterFlex ChemDurance Pump Tubing, 06442-14, #14), purchased from Cole-Parmer Instrument Company Ltd., St Neots. Once electrolyte flow was started through the redox-

flow battery cell, the electrolytes were degassed rigorously using nitrogen gas for 30 min. After degassing, all needles were removed from the septum at the top of the reservoir and parafilm was used to improve the seal on the reservoirs. This approach was found to be favourable when using the in-house nitrogen supply, which contains small amounts of oxygen that can continuously re-oxidise 2,6-$DHAQ^{4-}$ throughout the experiment if constantly supplied. It is recommended to use pure cylinder nitrogen if continuous nitrogen flow is needed throughout the experiment.

A portable potentiostat (BioLogic SP-150) was used to measure the cell voltage throughout the experiment, with a time interval of 1 s between datapoint collections. The potentiostat was used for open-circuit voltage measurements during rest periods, or to charge (and discharge) the battery between cell voltage of 0.6 V and 1.5 V (using a constant current of 50, 25 or 10 mA). When using 10 mA, the constant-current charge was cut off at 10 h (Figure S5 and Figure S6), to minimise the degradation of 2,6-DHAQ that occurs in the catholyte and anolyte at high voltages.[1] Schematic diagrams and photographs of the redox-flow battery cell are given in Figure S12.

Methods S3: On-line NMR apparatus

A commercial NMR flow tube was used for on-line detection (InsightMR 2.0, Bruker, see Supplementary Figure S13 and Figure S14). The inlet and outlet tubing (1/16” OD PFA) was inserted into the catholyte-side electrolyte reservoir via the septum at the top. The catholyte was pumped through the flow tube at 6 rpm (~1.6 mL min^{-1}) using a third peristaltic pump (MasterFlex L/S Pump, 07528-30; Easy-load Pump Head, 77200-60; MasterFlex ChemDurance Pump Tubing, 06442-14, #14). The flow tube was filled with the catholyte from the reservoir and placed in the spectrometer before flow was initiated through the redox-flow battery cell. This was to allow time for NMR optimisation (shimming, referencing, tuning, pulse calibration), before the crossover experiment was started. The 400 MHz (9.4 T) wide-bore NMR spectrometer was equipped with a BBO 400 MHz W1S2 5mm Z-gradient diffusion probe (Bruker). All the redox-flow battery equipment was placed outside the 5 gauss (G) line.

Methods S4: NMR acquisition

Pseudo-2D ^{1}H NMR experiments were performed by direct excitation with a 90° pulse. The 90° pulse length was between 18.25-18.38 μs for every experiment. Pulse calibration was performed at different battery state-of-charge, and any changes to the 90° pulse widths were found to be insignificant. Each spectrum was obtained by using 8 scans, a 7.50 s acquisition time, and 30 s recycle delay (d1), resulting in a spectrum recorded every 5 min ((7.50 s + 30 s) × 8 = 300 s). The d1 chosen was quantitative for oxidised 2,6-DHAQ H_A throughout battery charging (see Tables S1-3 and Figure S4), as increasing the

d1 did not change the integration of the signal. 2,6-DHAQ H_A was used for quantification because it has the shortest longitudinal relaxation time (T_1), therefore maximising the potential temporal resolution of the experiment. Furthermore, it does not undergo H-D exchange.[1] The NMR acquisition was started before the flow of electrolyte through the redox-flow battery cell was initiated, so that ^{1}H NMR spectra were being collected from the very start of the crossover experiment. All spectra were referenced to the chemical shift 4.8 ppm using an external D_2O reference. The probe temperature was set to 25 ^{o}C for all experiments. The room temperature was kept between 20-21 ^{o}C.

Methods S5: Quantitative NMR

To ensure that the spectra were quantitative for oxidised 2,6-DHAQ H_A, inversion-recovery experiments were used to measure the T_1 for these protons in the catholyte. The t1ir pulse sequence implemented on Bruker Topspin software, consists of 180° and a 90° pulses, with an increasing delay time (*t*) between the two pulses for each spectrum in the second dimension. Eight spectra were acquired in the second dimension, using a d1 that is quantitative for 2,6-DHAQ proton A (d1 = 30 s > T_1 * 5).

As the T_1 of DHAQ protons depends on the state-of-charge of the catholyte that 2,6-DHAQ is dissolved in, the inversion-recovery experiments were repeated in catholyte solutions at 0%, 50% and 100 % state-of-charge. Each catholyte was prepared by changing the ratio of ferrocyanide and ferricyanide added to the supporting electrolyte. The catholytes (20 mL) were then spiked with 0.1 M 2,6-DHAQ anolyte, so that the resulting 2,6-DHAQ concentration was 0.002 M. The T_1 of 2,6-DHAQ H_A was found to be 2.27 ± 0.05 s, 1.85 ± 0.02 s and 1.47 ± 0.03 s in the 0%, 50% and 100% state-of-charge catholytes, respectively (Supplementary Table S1). These experiments were also repeated with different concentrations of 2,6-DHAQ, and KOH, to simulate the expected changes in concentrations throughout the crossover experiment, as summarised in Tables S2 and S3.

Once the quantitative d1 (d1 > $T_1(H_A)$ *5) was determined, the "quantitative flow rate regime" was found by collecting ^{1}H NMR spectra of these 2,6-DHAQ-spiked catholytes at different flow rates (between 0 and 5 mL min^{-1}) and defined as the flow rate at which no signal suppression is observed as a result of electrolyte flow (Figure S4). To further confirm that no flow-rate dependant signal suppression was observed during the experiments, stopped-flow experiments were carried out at different points during the charge discharge cycle. No signal suppression due to flow was observed throughout the experiment.

It is important to note that the catholyte residence time within the NMR flow tube is complicated by fluid dynamics. Under these conditions, it is likely that there will be laminar flow of the catholyte and some degree of back-mixing, which will increase the average residence time. A. M. R. Hall *et al.*[2] observed non-ideal plug flow in the tip end of the InsightMR tube, which leads to an increase distribution in fluid residence times. This is one of the reasons it is important to use experimental methods to determine our quantitative flowrate and NMR parameters (Tables S4, Figure S4 and Figure S6), to ensure that impact of variable

sample residence times and changes in magnetic field experienced by the sample before reaching the detection region were accounted for. The non-ideal flow in the NMR tube is unlikely to have a significant influence when measuring slow processes such as crossover, as we are observing changes over tens of minutes to hours. However, this flow behaviour could cause noticeable delays in the response times when measuring processes that occur on the order of seconds to minutes.

Methods S6: Calibrating 2,6-DHAQ concentration

The signal intensity was calibrated with a series of 2,6-DHAQ calibrations solutions in 1 M KOH (0.05 M, 0.025 M, 0.01 M, 0.005 M, 0.0045 M, 0.00226 M and 0.00045 M 2,6-DHAQ). A one-pulse ^1H NMR spectrum was taken for each solution using a d1 = 70 s, so that they were quantitative for H_A, H_B and H_C. All other NMR acquisition parameters were kept the same as those used in the on-line crossover experiments. The volume of the NMR flow tube is slightly lower than that of a standard 5 mm solution-state NMR tube. Therefore, for the 0.002 M 2,6-DHAQ calibration solution, a quantitative ^1H NMR spectrum was also taken in the flow tube apparatus, and a volume correction factor was calculated from the ratio of peaks between the two spectra. The calibration was then corrected for the lower volume in the flow tube apparatus.

In all of these experiments, the 2,6-DHAQ concentration in the catholyte side was calculated by quantifying proton A of oxidised 2,6-DHAQ (2,6-$DHAQ^{2-}$). When 2,6-$DHAQ^{2-}$ passes through the membrane at low state-of-charge, it is not reduced to $DHAQ^{4-}$ by the excess of ferrocyanide, because this is thermodynamically unfavourable (ΔE^{Θ} = $\Delta E^{\Theta}_{reduction}$ – $\Delta E^{\Theta}_{oxidation}$ = (-0.68 V vs SHE[3]) – (0.51 V vs SHE[3]) = -1.19 V). Conversely, at higher state-of-charge, when reduced 2,6-DHAQ (2,6-$DHAQ^{4-}$) crosses through the membrane into the catholyte side, it is oxidised to 2,6-$DHAQ^{2-}$ by the excess of ferrocyanide present (ΔE^{θ} = $\Delta E^{\Theta}_{reduction}$ – $\Delta E^{\Theta}_{oxidation}$ = (0.51 V vs SHE) - (-0.68 V vs SHE) = +1.19 V). During the course of the experiment, no 2,6-$DHAQ^{4-}$ proton resonances were detected in the catholyte side. As the chemical shift changes with battery state-of-charge, assignment of 2,6-$DHAQ^{2-}$ vs 2,6-$DHAQ^{4-}$ was made by comparing peak separation between H_A and H_B, which is ~1.1 ppm and ~1.5 ppm for 2,6-$DHAQ^{2-}$ and 2,6-$DHAQ^{4-}$, respectively[1] (Figures S7 and S8).

Methods S7: Determining the battery state-of-charge using on-line NMR

The state-of-charge of the battery was monitored by measuring the change in HOD chemical shift during the experiment. As ferrocyanide (d^6, high-spin, diamagnetic) is converted to ferricyanide (d^5, high-spin, paramagnetic) during charging, the change in radical concentration changes the bulk magnetism of the solution. The change in bulk magnetism leads to an increase in chemical shift of all species in solution; the Evans method therefore can be used to calculate the radical concentration from this shift.[4,5] As the

state-of-charge is proportional to radical concentration, this phenomenon can be exploited to monitor the state-of-charge of the battery.[4] To determine exactly how this change in chemical shift relates to state-of-charge, a series of catholyte solutions with different ferrocyanide:ferricaynide ratios where prepared, and their chemical shift measured. There is a linear relationship between ferricyanide concentration and the chemical shift of the HOD catholyte, as shown in Figure S9. This linear relationship was then used to calculate the concentration of ferricyanide from the HOD chemical shift throughout the crossover experiments. The battery capacity is limited on the anolyte side, so the change in concentration of ferricyanide is scaled relative to the starting capacity in the 2,6-DHAQ anolyte to give the state-of-charge. Note that 2,6-$DHAQ^{2-}$ accepts two electrons per molecule, whereas ferrocyanide accepts one. Therefore, the measured change in ferricyanide concentration in the catholyte is halved to give the estimated 2,6-$DHAQ^{4-}$ concentration in the anolyte. State-of-charge is given by the percentage ratio of 2,6-$DHAQ^{4-}$ relative to the starting amount of 2,6-$DHAQ^{2-}$. As the starting catholyte already contains some ferricyanide, as is the general practice in ferrocyanide flow batteries, there is a slight drop in measured state-of-charge at the start of the experiment due to ferricyanide degradation,[6,7] reduction[8] and crossover.

Methods S8: Setup of the on-line NMR crossover studies

The setup of the redox-flow battery and NMR is described in the Methods sections above and detailed in Figure S12-15. For electrolyte level monitoring, a snapshot image of the electrolyte reservoir levels was captured for both electrolyte reservoirs every 5 min. The changes in electrolyte volumes were monitored and found to be insignificant (see Figure S16-S19). Prior to starting the flow through the redox-flow battery cell, the catholyte was flowed through the flow NMR apparatus, so that the NMR acquisition parameters could be optimised ahead of the crossover experiment. Once the NMR was setup, the pseudo-2d NMR and electrochemical data acquisition was started simultaneously. After 15 min (after three background ^{1}H NMR spectra had been collected), the outlets to the electrolyte reservoirs were then opened and the electrolytes were pumped through the redox-flow battery cell. The time at which the electrolytes first reach the redox-flow battery cell was recorded at the start of the crossover experiment. After a further 15 min (or a further three ^{1}H NMR spectra), electrolyte degassing was started. Finally, after 30 min degassing (six ^{1}H NMR spectra), the nitrogen inlet and outlet needles were removed, and parafilm was used to improve the seal at the top of the reservoirs. After this point, no further changes were made to the apparatus. A light was kept on in the room at all times during the experiment, to allow for continuous monitoring of the electrolyte levels via a webcam at constant light conditions. The resulting ^{1}H NMR spectra were processed in TopSpin then analysed using MATLAB.

At the flow rate of 16 mL min^{-1}, the electrolytes took <10 s to be pumped from the electrolyte reservoir, through the redox-flow battery cell and returned to the reservoir. At the flow rate of 1.6 mL min^{-1}, the catholyte takes 3.5 min to be transferred from the bottom of the reservoir to the bottom of the InsightMR flow tube, and 2.7 min to be returned to the reservoir again. A higher flow rate was used to supply the

redox-flow battery because it was optimal for battery operation and also allowed for fast mixing in the electrolyte reservoirs. Assuming there was complete mixing in the reservoirs, the NMR spectra collected therefore represent an average of the system over a 5 min period, with a 3.5 min delay after leaving the electrochemical cell. The flow rates for the redox-flow battery and NMR apparatus were calibrated by recording the time taken for a given volume (1-5 mL) of electrolyte to be transferred into a measuring cylinder at a range of flow rates, as is summarised in Supplementary Table S4.

Methods S9: Permeability, crossover rate and error determination

The permeability and crossover rates were determined by performing a linear fit of the change in 2,6-DHAQ concentration according to Equation S1 and S2, respectively. The linear fits were performed on MATLAB using *polyfit*, and 95% confidence limits were used as the error estimation for the permeability and crossover rate. The estimated amount of 2,6-DHAQ crossover (mol) was calculated for each charging period by multiplying the crossover rate by the membrane area (m^2) and time per charge (h). The same calculation was performed on the error in crossover rate, to give the error in moles of 2,6-DHAQ. The estimated background crossover shown in Figure 4b is plotted as a function of the average of the background crossover rate measured in Cell 2 and 3 during the first 12 h before battery charging. The upper and lower limit of the estimated background crossover given is the upper and lower 95% confidence limit for background crossover in Cell 2 and Cell 3, respectively.

Table S1: Measured $T_1(H_A)$ of 0.002 M 2,6-DHAQ^{2-}, prepared in catholyte solutions with varying ferrocyanide and ferricyanide concentrations, representative of solutions between 100% and 0% state-of-charge. The supporting electrolyte for each catholyte solution was 1 M KOH in D_2O.

T_1 of 2,6-DHAQ H_A / s	Concentration of potassium ferrocyanide trihydrate / M	Concentration of potassium ferricyanide / M
2.27 ± 0.05	0.250	0.040
1.85 ± 0.02	0.145	0.145
1.47 ± 0.03	0.000	0.290
2.68 ± 0.02	0.000	0.000

Table S2: Measured $T_1(H_A)$ for 2,6-DHAQ^{2-} in the catholyte solution at different concentrations. The catholyte solution used was prepared the same as it was for on-line crossover experiments (0.25 M ferrocyanide with 0.04 M ferrocyanide in 1 M KOH/D_2O).

T_1 of 2,6-DHAQ H_A / s	2,6-DHAQ / M
2.27 ± 0.05	0.002
2.25 ± 0.06	0.001

Table S3: Measured $T_1(H_A)$ of at 0.002 M 2,6-DHAQ^{2-} in different KOH concentrations. The solutions were prepared in 0.29 M potassium ferricyanide with either 1.0 or 1.2 M KOH with D_2O solvent. This represents the maximum OH^- concentration changes expected it OH^- was acting as the only as the charge-balancing species.

T_1 of 2,6-DHAQ H_A / s	Concentration of KOH / M
1.47 ± 0.03	1.0
2.1 ± 0.2	1.2

Table S4: Calibration data for the peristaltic pumps. Relationship between peristaltic pump rotations per minute (rpm) and electrolyte flowrate was found as described in Methods.

Flow rate / rpm	Flow rate / mL min^{-1}
16	4.62
14	4.14
12	3.24
10	3.00
8	2.26
6	1.56
4	1.07
2	0.63
1	0.30
0	0.00

Equation S1: Fick's first law used to calculate permeabilities, where *P* is the permeability (cm^2 s^{-1}), *l* is membrane thickness (cm), *t* is permeation time (s), *V* is permeate volume (cm^3), *A* is the accessible membrane surface area (cm^2), and C_p and C_f are the concentrations of DHAQ in the permeate and feed respectively (both in mol cm^{-3}).

$$P = \frac{VC_p l}{tAC_f}$$

Equation S2: Equation used to calculate crossover rate in mol m^{-2} h^{-1}, where *t* is time (h), *A* is the accessible membrane surface area (m^2), and n_{DHAQ} is the amount of 2,6-DHAQ in the catholyte side (mol).

$$Crossover\ rate\ (DHAQ) = \frac{n_{DHAQ}}{tA}$$

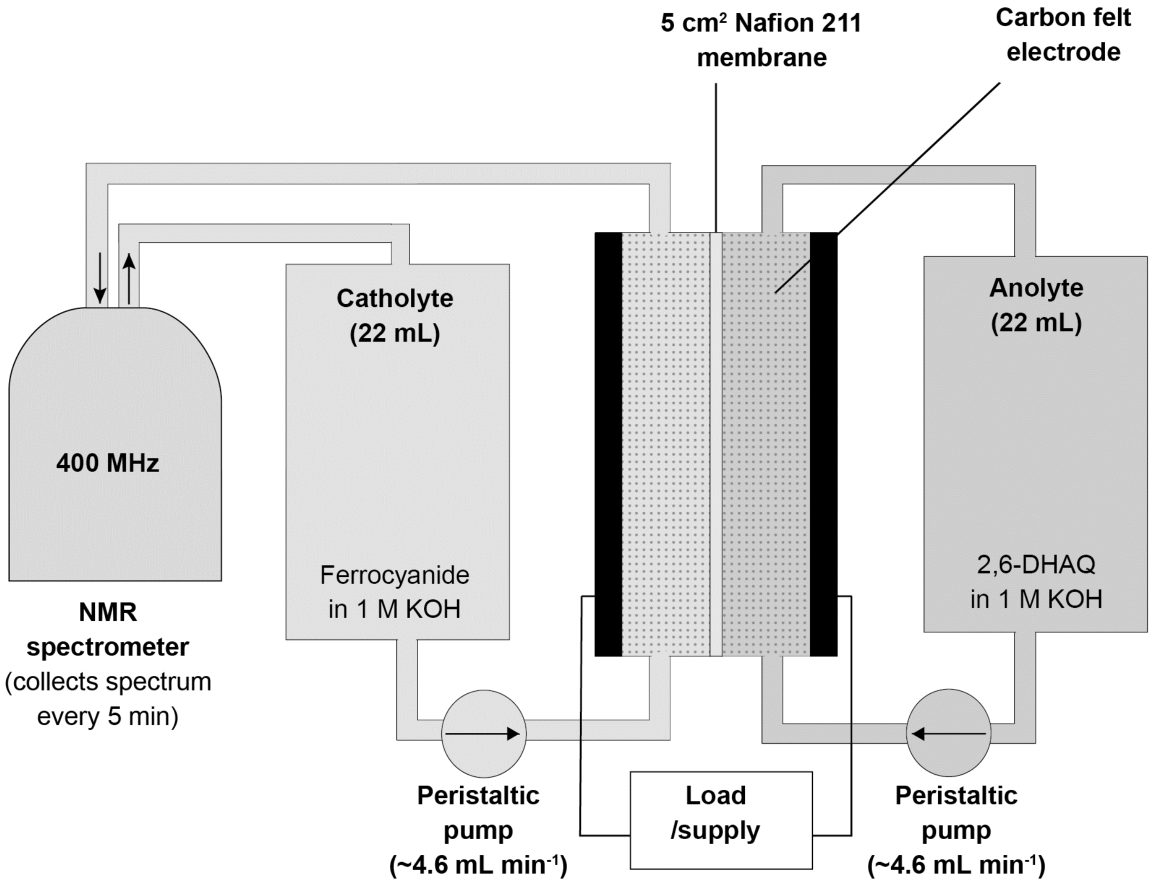

Figure S1: Schematic for the two-pump on-line NMR setup used in earlier experiments. A two-pump setup, similar to those reported previously, where the electrolyte flow rates through the redox-flow battery and NMR apparatus are the same. When using this setup, a compromise is needed for both the NMR spectra quantitative and redox-flow battery efficiency. Stopped-flow experiments can be used to correct for NMR signal suppression that occurs at higher flow rates[2], though this is complicated by the changing T_1 of species throughout the experiment (Tables S1-3), which will also change the degree of signal suppression (Figure S4). One of the advantages of this setup, is that less pumps are required, and the electrolyte is transferred directly from the electrochemical cell to the NMR spectrometer. This setup is therefore better suited to detecting short-lived species formed in the electrochemical cell, as the transfer time to the NMR is shorter.

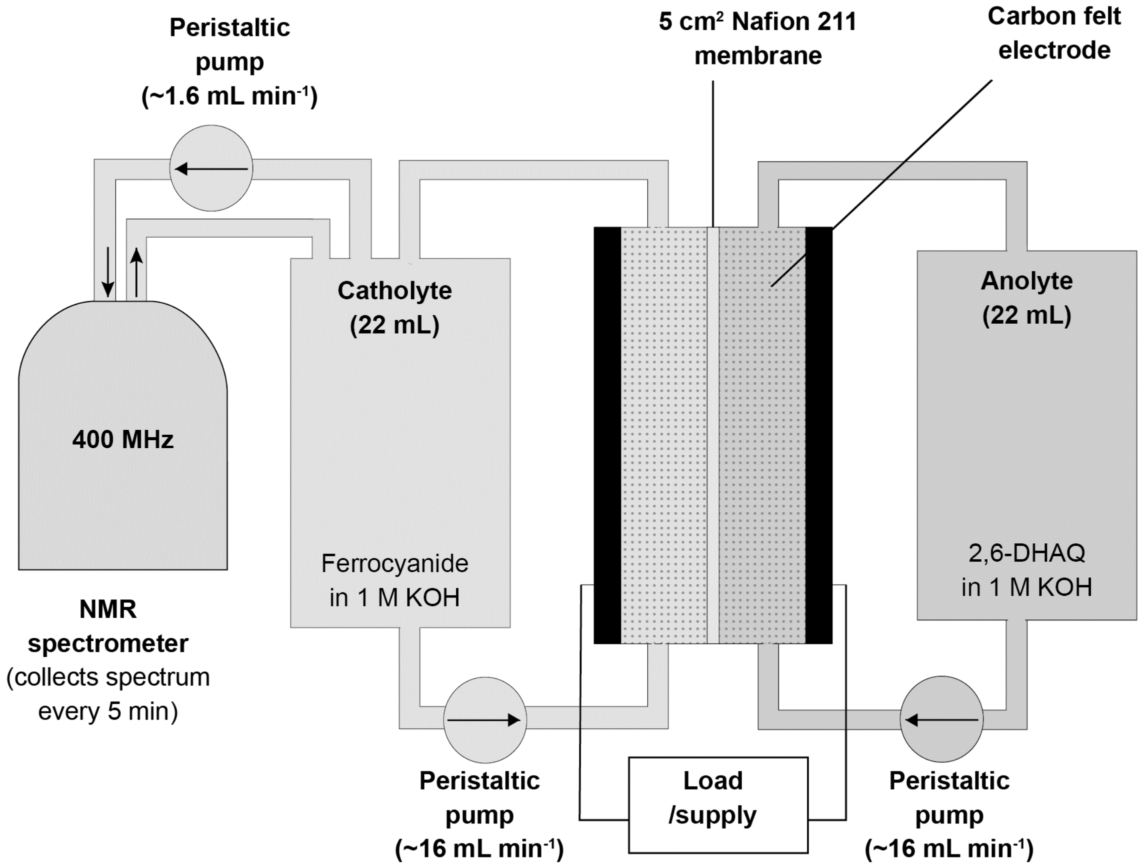

Figure S2: Schematic for the three-pump on-line NMR setup used in our crossover experiments. The two pumps supplying the redox-flow battery are set to a flow rate that is optimal for the electrochemistry (~16 mL min^{-1}). The third pump supplying the on-line NMR is set to ~1.6 mL min^{-1}, which sufficiently low for the ^{1}H NMR spectra to be quantitative for 2,6-DHAQ H_A. The flow can be started through the NMR spectrometer before starting flow through the redox-flow battery cell, meaning that there is time to setup NMR parameters (e.g. shimming and pulse calibration), before starting the crossover experiment. Greater signal-to-noise can also be achieved with the three-pump setup, as the quality of the NMR spectra improves as the flow rate is decreased, making it well suited to crossover experiments. This configuration is also favoured for any NMR experiments where stopped flow is required (e.g. Diffusion Ordered SpectroscopY), as the NMR flow can be stopped whilst the redox-flow battery keeps operating.

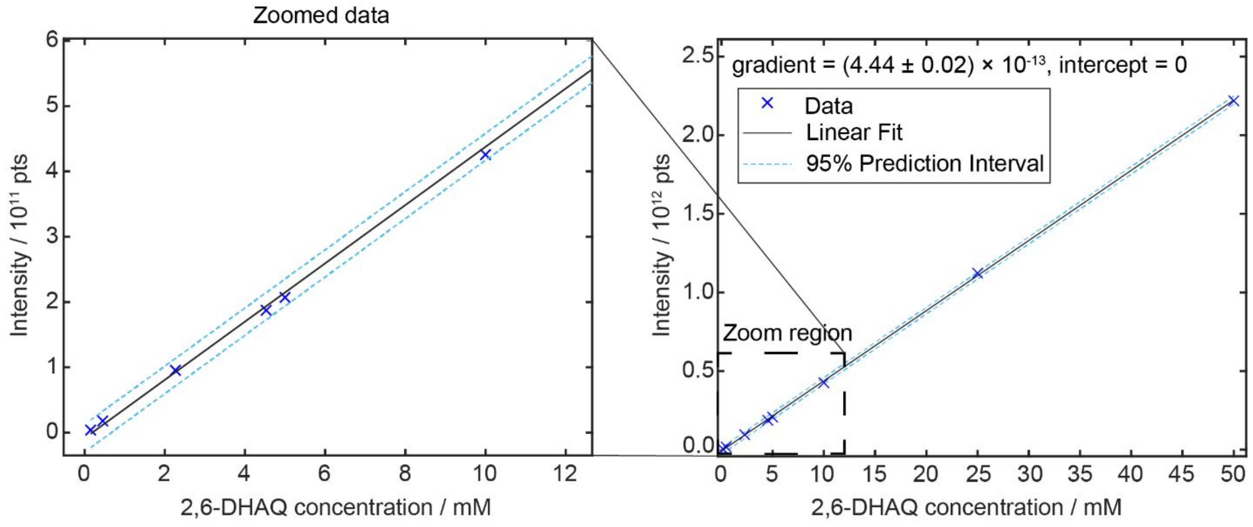

Figure S3: Calibration curves for 2,6-DHAQ H_A, with zoomed data in the low concentration region shown on the left. The signal intensity is volume corrected to account for the smaller sample volume in the detection region for the flow NMR tube compared to the standard static 5 mm NMR tubes used for calibration. The volume correction factor was measured by comparing the signal intensity of the HOD peak for identical calibration solutions analysed in the InsightMR tube (in the absence of flow) and then in the standard 5 mm NMR tube used for calibration.

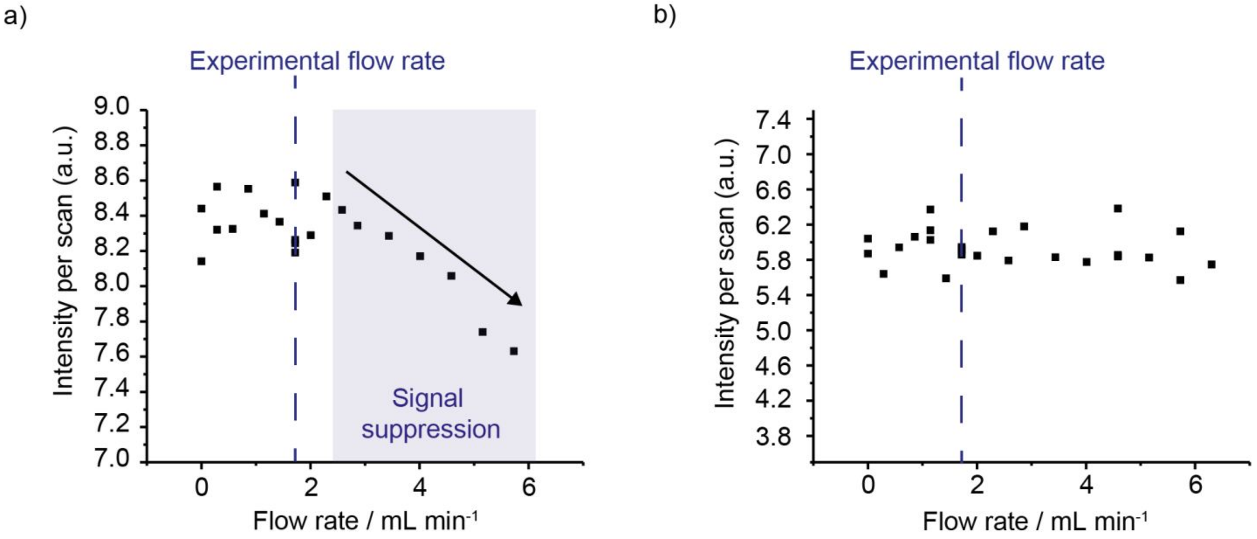

Figure S4: Flow signal suppression experiments. Plot showing the signal intensity of 2,6-DHAQ H_A as a function of NMR supply flowrate in a) the catholyte solution as prepared for crossover experiments before charging (0.25 M ferrocyanide with 0.04 M ferricyanide in 1 M KOH) and b) a ferricyanide solution, replicating the maximum ferricyanide concentration that could be seen at 100% state-of-charge (0.29 M ferricyanide in 1 M KOH). The concentration of 2,6-DHAQ in each solution was 0.002 M.

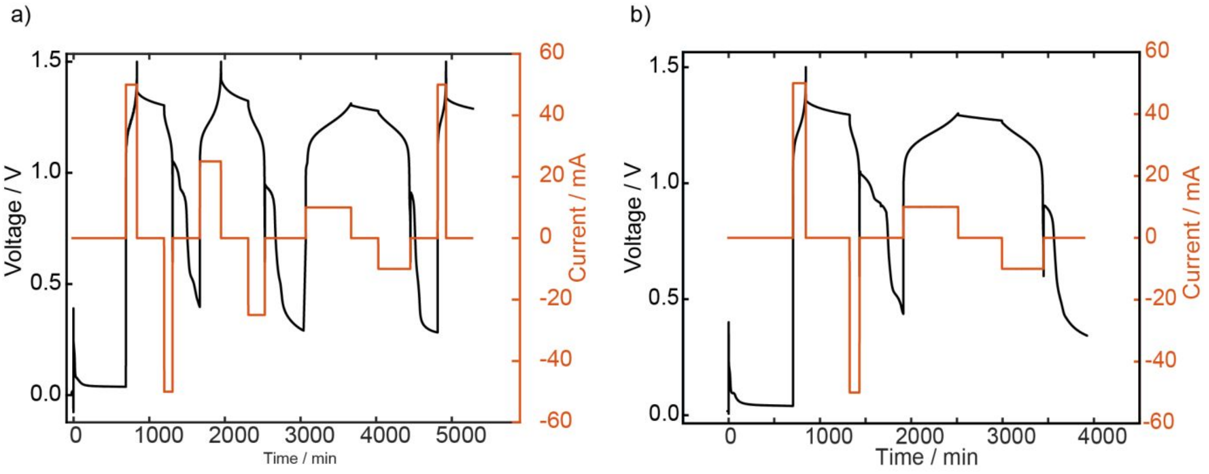

Figure S5: Applied current and measured cell voltage a) Cell 2 and b) Cell 3 during the crossover experiments.

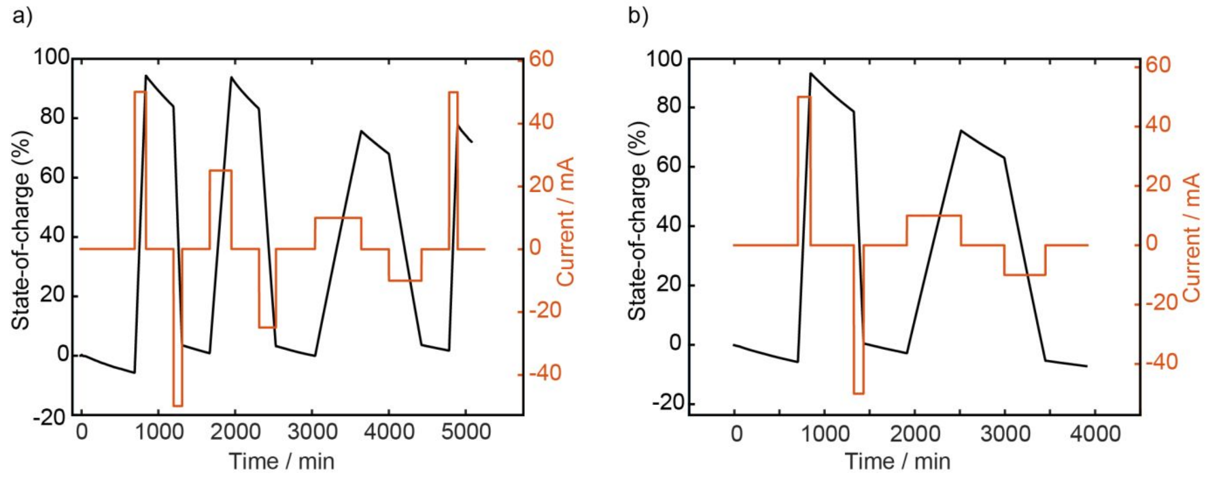

Figure S6: Applied current and measured state-of-charge for a) Cell 2 (protocol a) and b) Cell 3 (protocol b) during the crossover experiments. The state-of-charge was determined from the chemical shift of HOD (see Methods S7 and Figure S7).

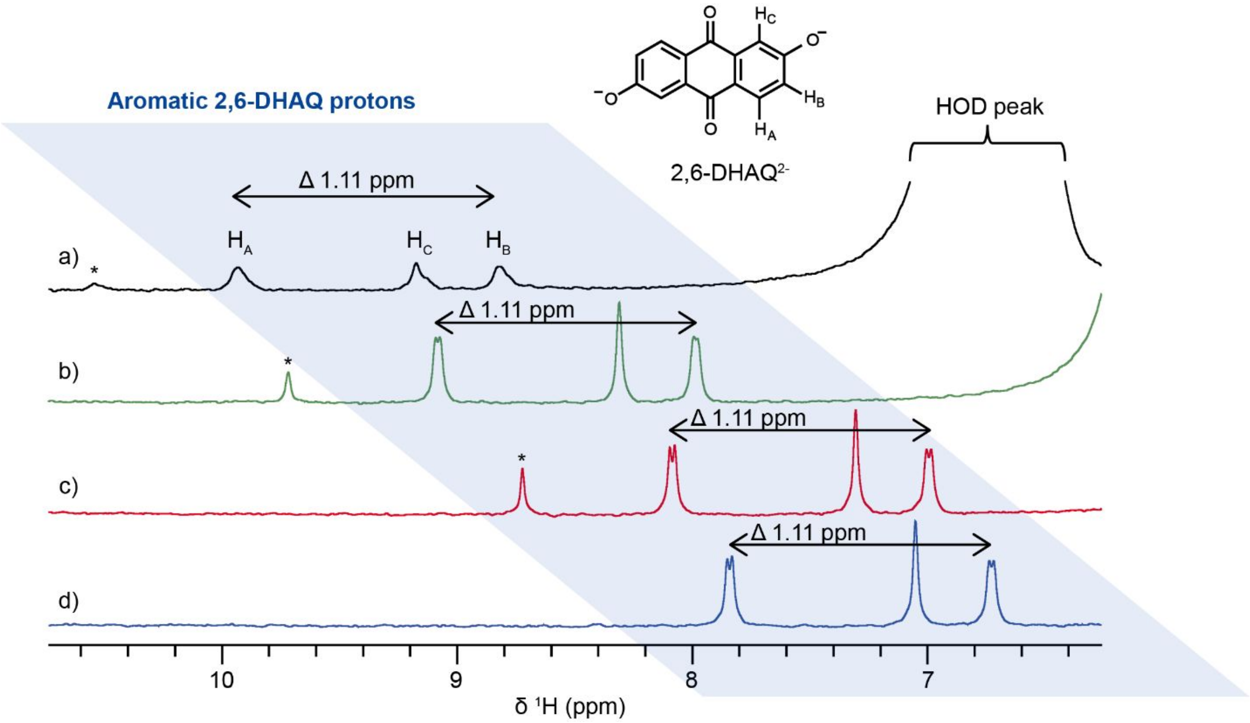

Figure S7: ^{1}H NMR of 0.002 M 2,6-DHAQ^{2-} prepared in electrolytes replicating a) 100% SOC catholyte (0.29 M ferricyanide), b) 50% SOC catholyte (0.145 M ferrocyanide and 0.145 M ferricyanide) and c) 0% SOC catholyte (0.25 M ferrocyanide and 0.04 M ferricyanide), all prepared in 1 M KOH with D_2O solvent. d) shows the ^{1}H NMR spectra 0.002 M 2,6-DHAQ^{2-} in the 1 M KOH electrolyte for comparison. Notably, the peak separation between H_A and H_B remains constant and is consistent with 2,6-DHAQ^{2-} literature assignments[9]. *Formamide impurities[10].

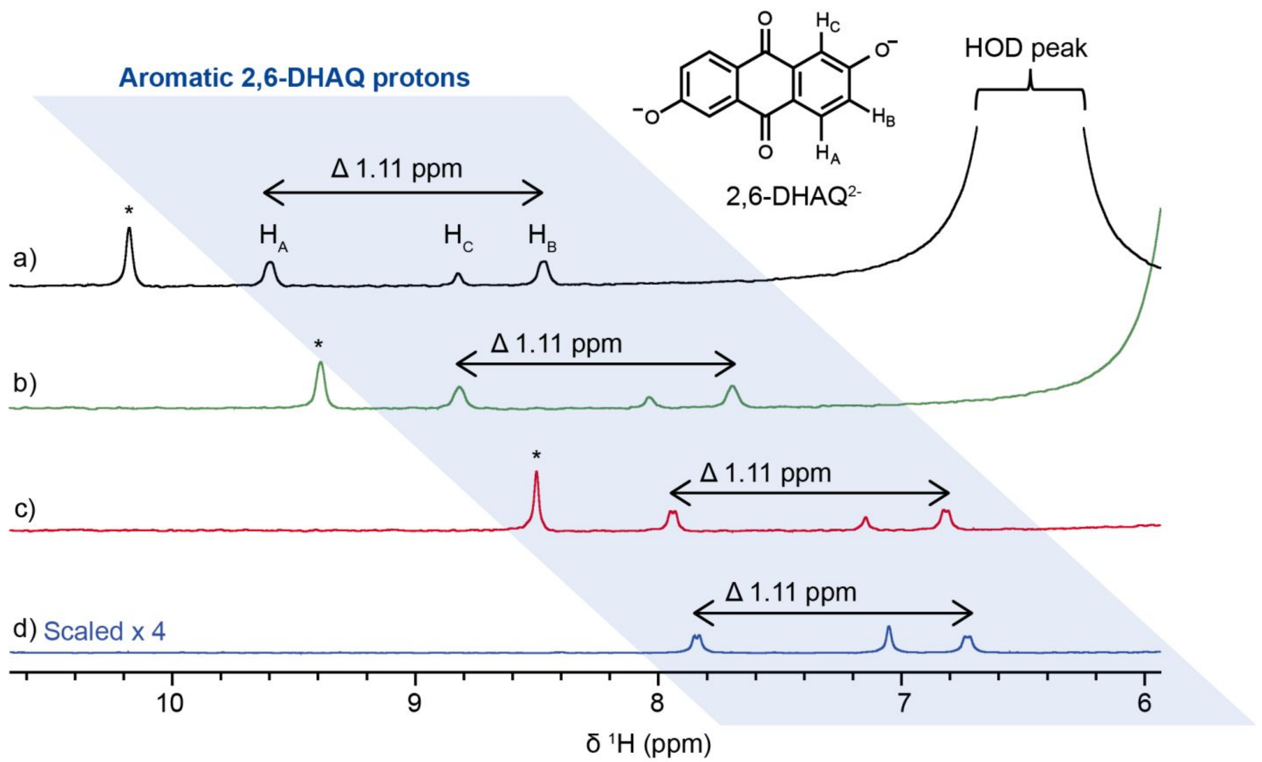

Figure S8: Selected on-line ^{1}H NMR spectra from the catholyte side of Cell 2 during a crossover experiment, at a) ~94% state-of-charge, b) ~50% state-of-charge and c) ~0% state-of-charge. d) Shows the ^{1}H NMR spectra 0.002 M 2,6-DHAQ^{2-} in the 1 M KOH electrolyte for comparison. Again, the peak separation between H_A and H_B remains constant and is consistent with 2,6-DHAQ^{2-} literature assignments[9]. Note that the loss in signal of H_c is a result of H-D exchange[9]. *Formamide impurities[10].

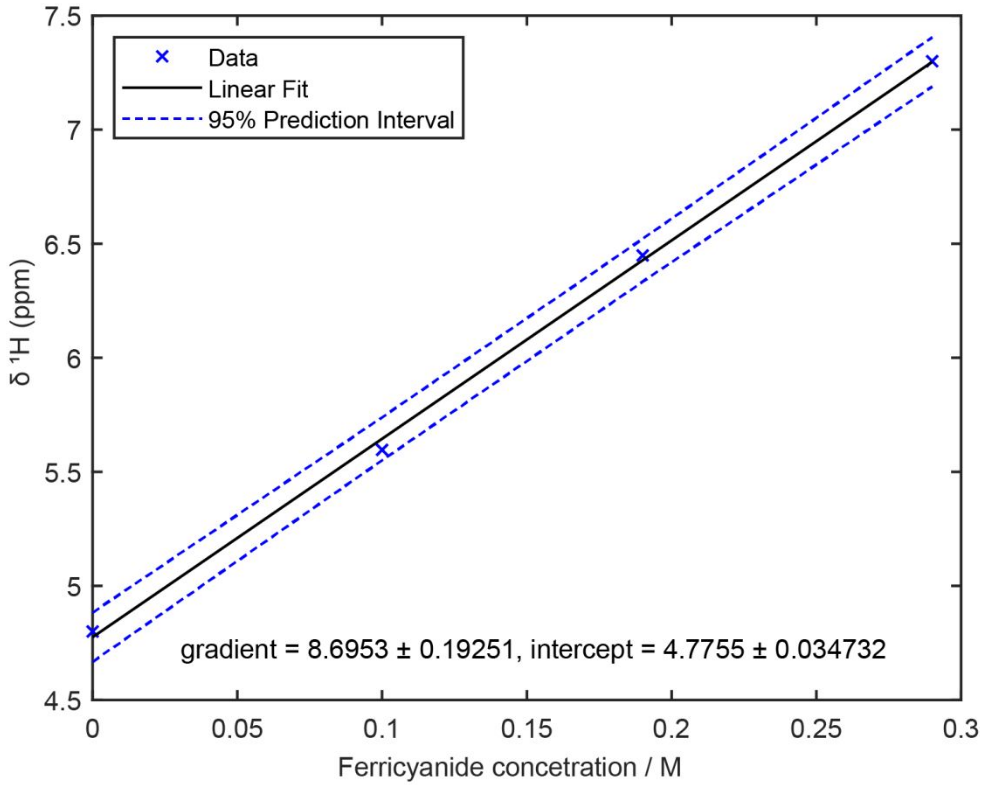

Figure S9: Plot showing the relationship between HOD chemical shift and ferricyanide concentration. The changes in chemical shift are proportional to the concentrations of paramagnetic species, which changes bulk magnetism of the solution[11]. This change in chemical shift can be related to the state-of-charge in the flow battery, as reported previously[4]. Note that for each solution, the total [ferricyanide + ferrocyanide] concentration was kept constant at 0.29 M, to replicate the concentrations that would be seen in the catholyte side during cycling.

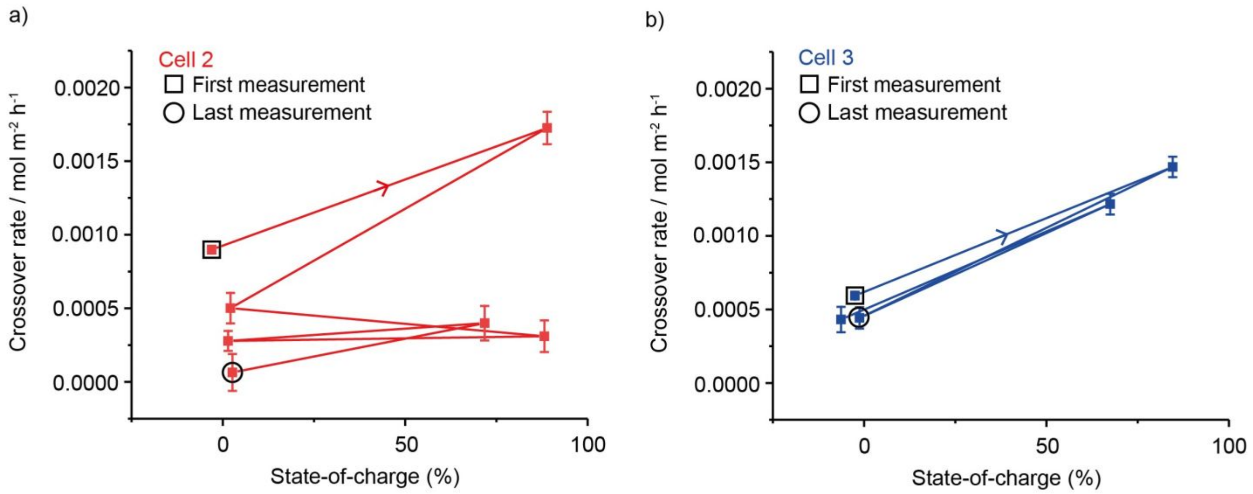

Figure S10: 2,6-DHAQ crossover rates plotted as a function of battery state-of-charge during cell rest. The order in which the rest periods took place is indicated with the arrows, and the first and last measurements indicated with a black square and circle, respectively. The data shown in a) corresponds to Cell 2, and b) to Cell 3.

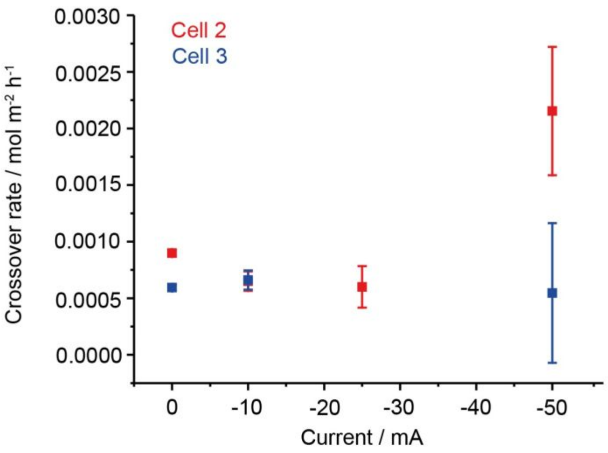

Figure S11: 2,6-DHAQ crossover rates plotted as a function of current during each constant-current discharge. Data for Cell 2 and 3 is plotted in red and blue, respectively.

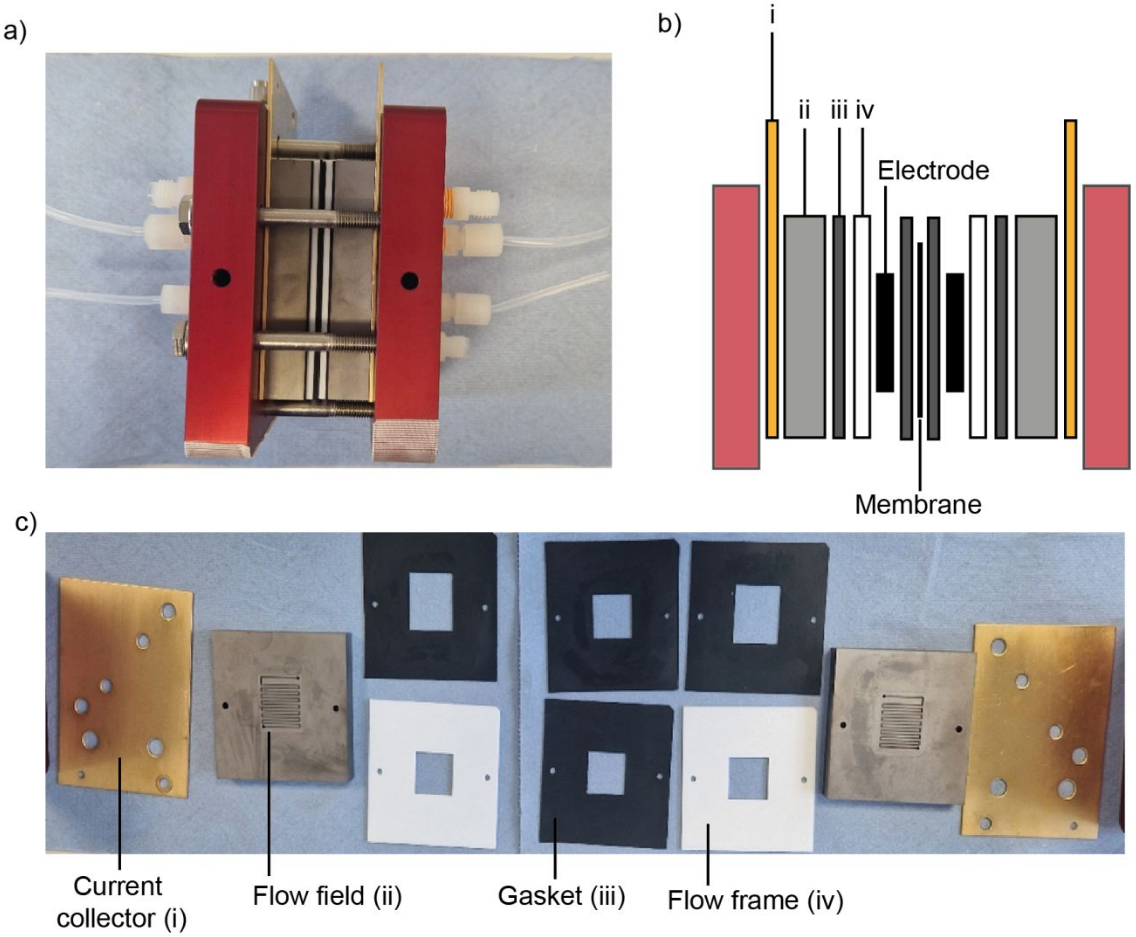

Figure S12: Setup of the redox-flow battery cell, shown with a) photograph of the assembled redox-flow battery cell, b) schematic diagram of the redox-flow battery cell and c) annotated photograph of the disassembled cell.

a) b)

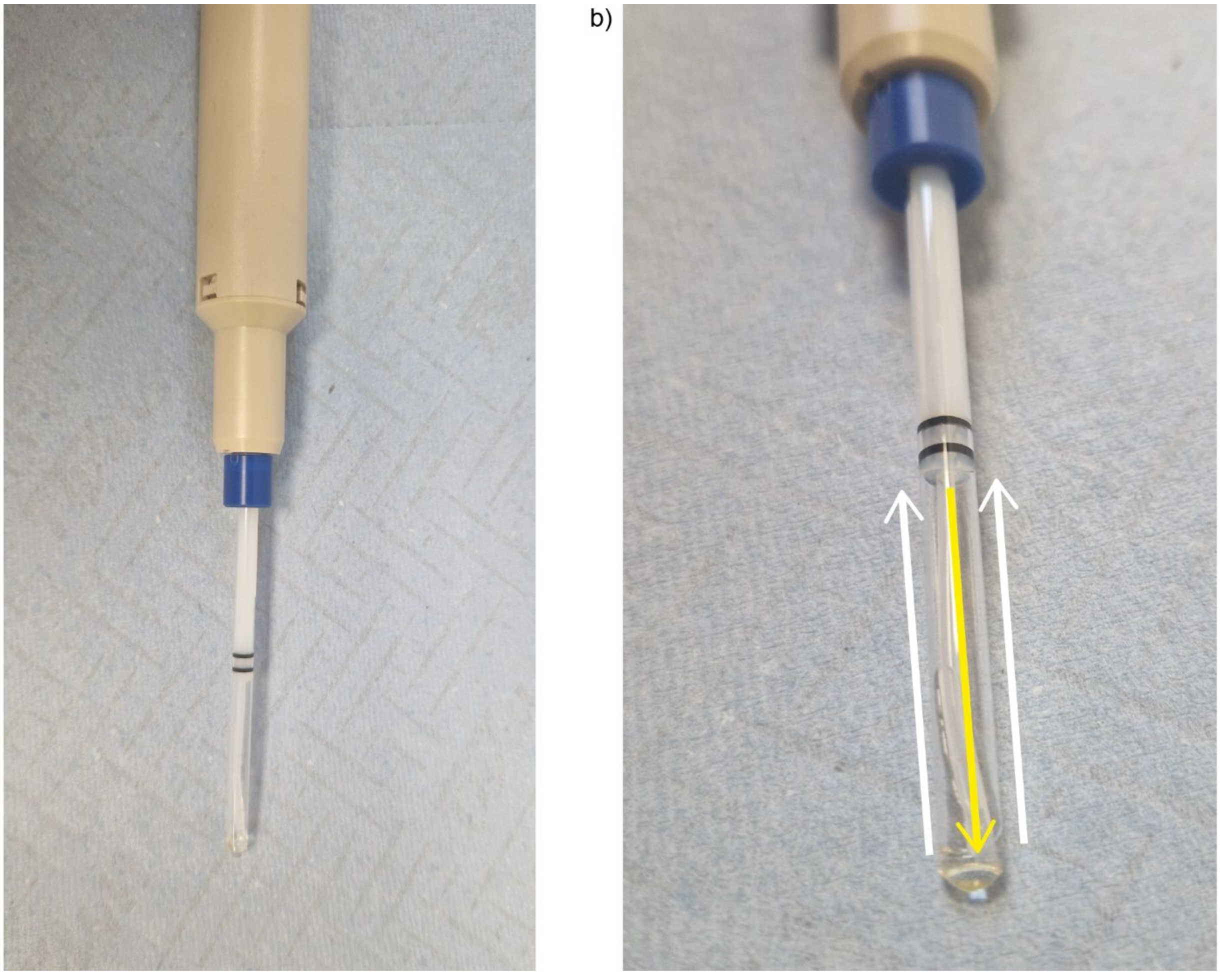

Figure S13: Annotated photograph of the Bruker InsightMR 2.0 flow NMR tube used for on-line NMR analysis. Note that the electrolyte flows into the bottom of the glass NMR tube via an inlet capillary tube (as indicated by the yellow arrow), and leaves through a hole at the top which is connected an outlet capillary (as indicated by the white arrows). The glass NMR tube is completely filled with sample when used during experiments.

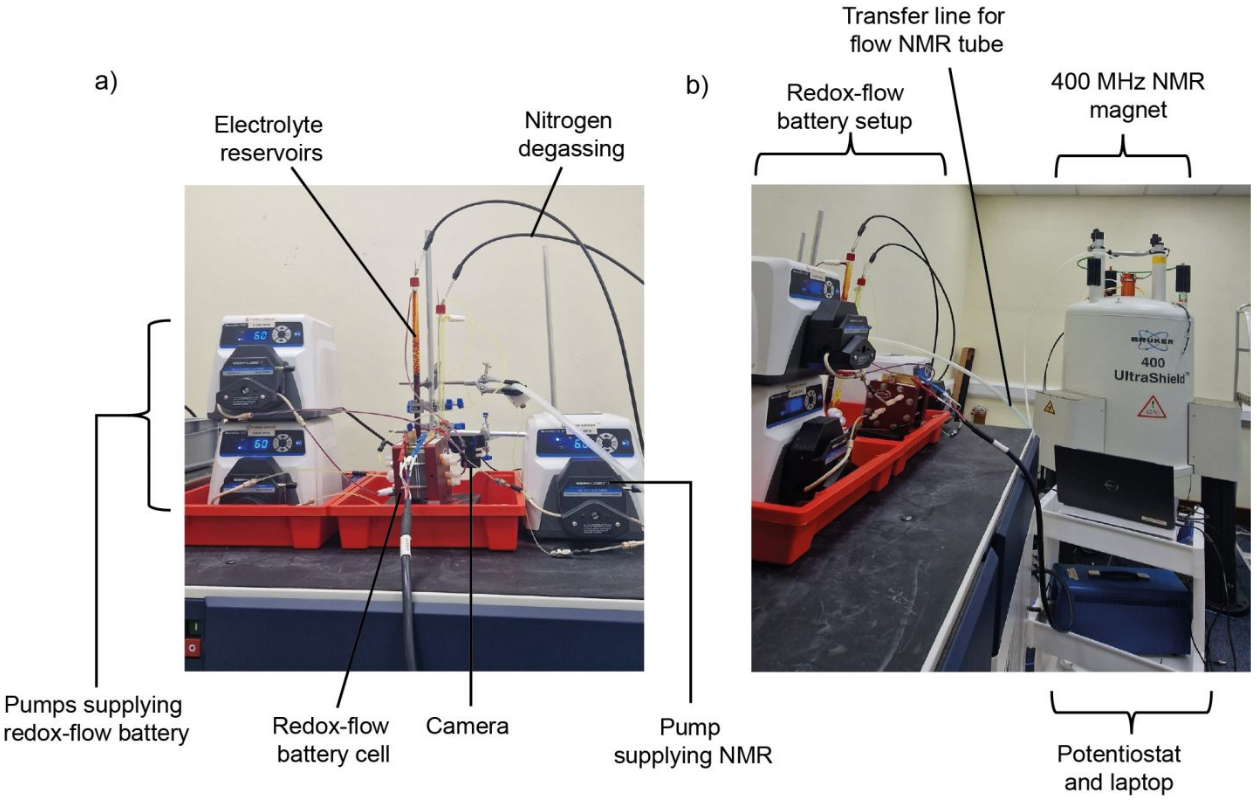

Figure S14: Annotated photographs of the on-line NMR crossover setup, where a) shows a close up of the redox-flow battery apparatus and b) shows the full experimental equipment setup.

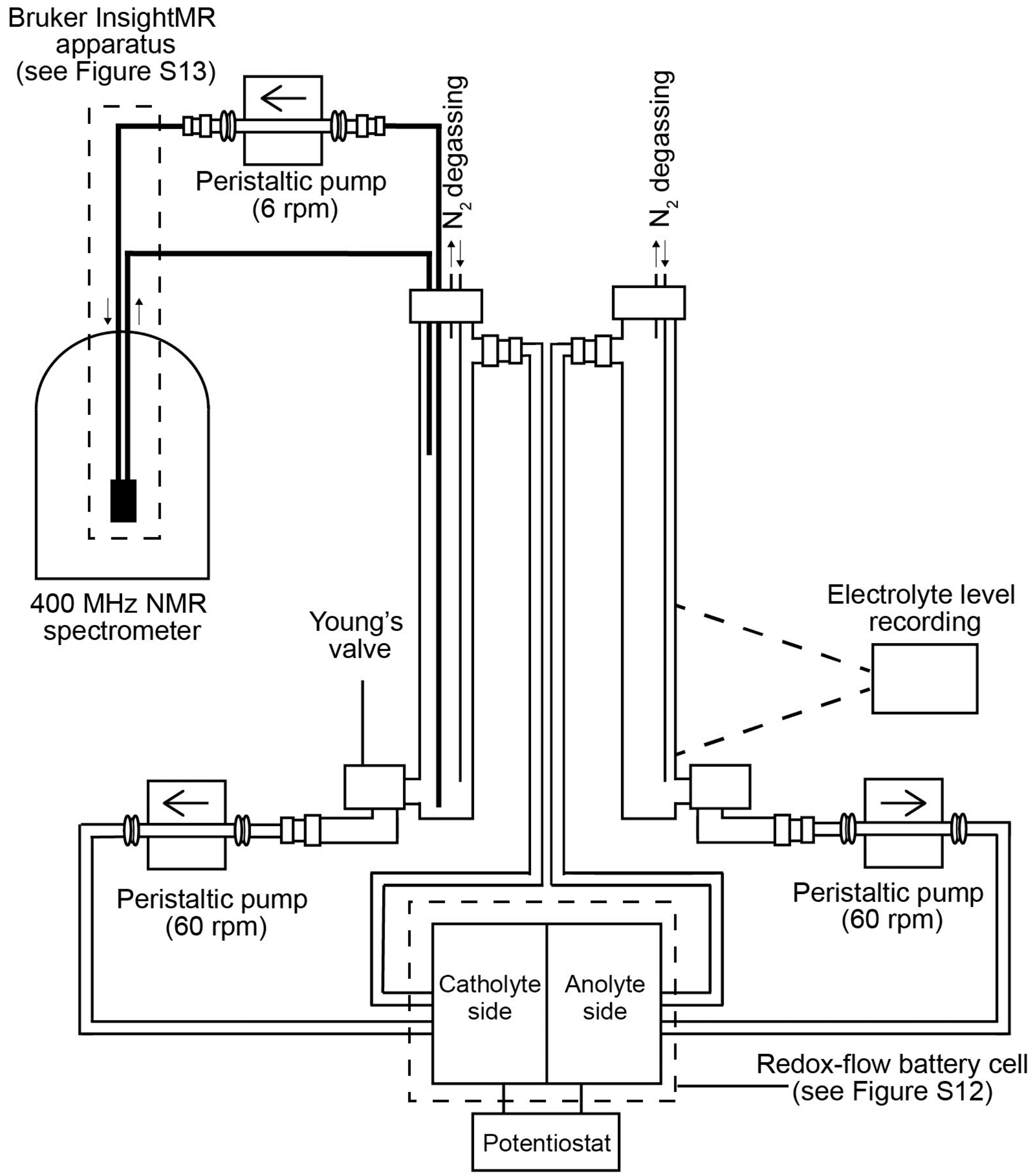

Figure S15: Schematic diagram of the whole on-line NMR crossover detection setup.

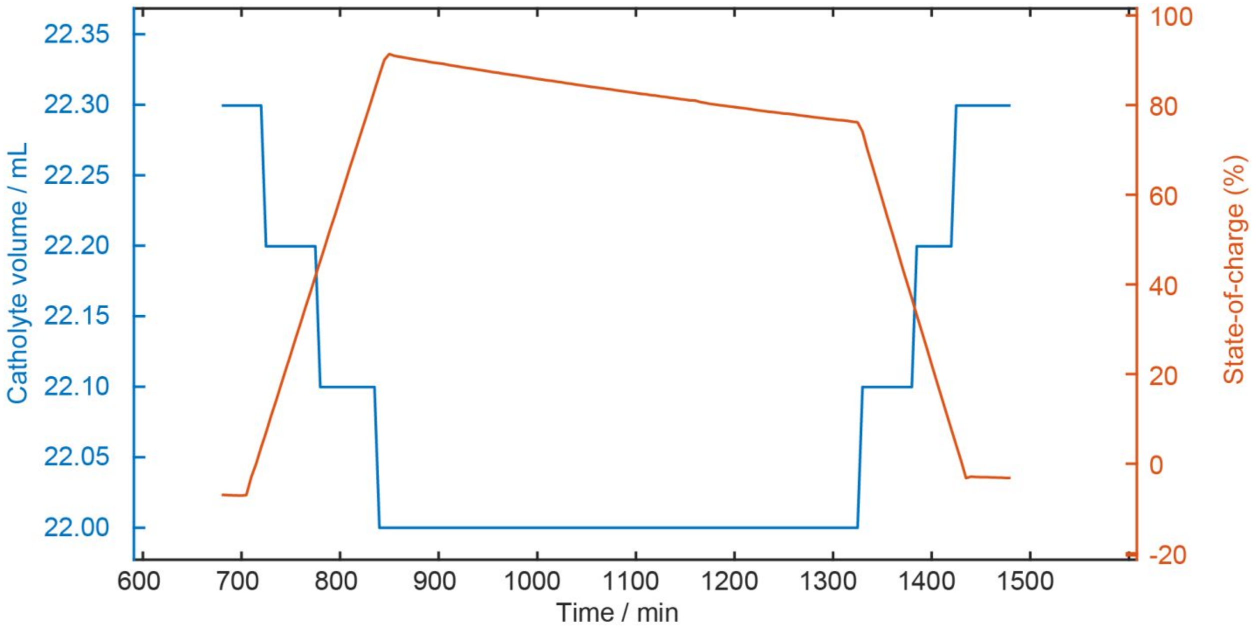

Figure S16: Plot showing measured catholyte level changes during one constant-current 50 mA charging cycle. The changes here are consistent with water crossover driven by electro-osmosis.

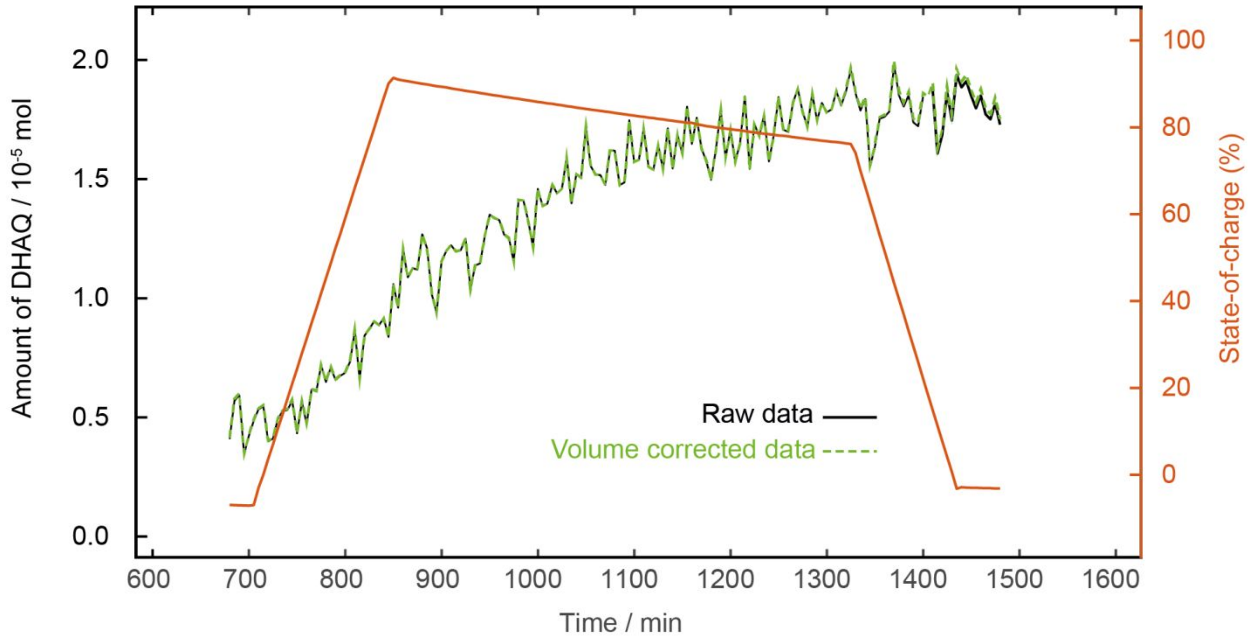

Figure S17: Impact of water crossover on 2,6-DHAQ crossover measurements during battery charging and discharging. The amount of 2,6-DHAQ crossover during cell rest is plotted in green and black for the volume corrected and raw data, respectively.

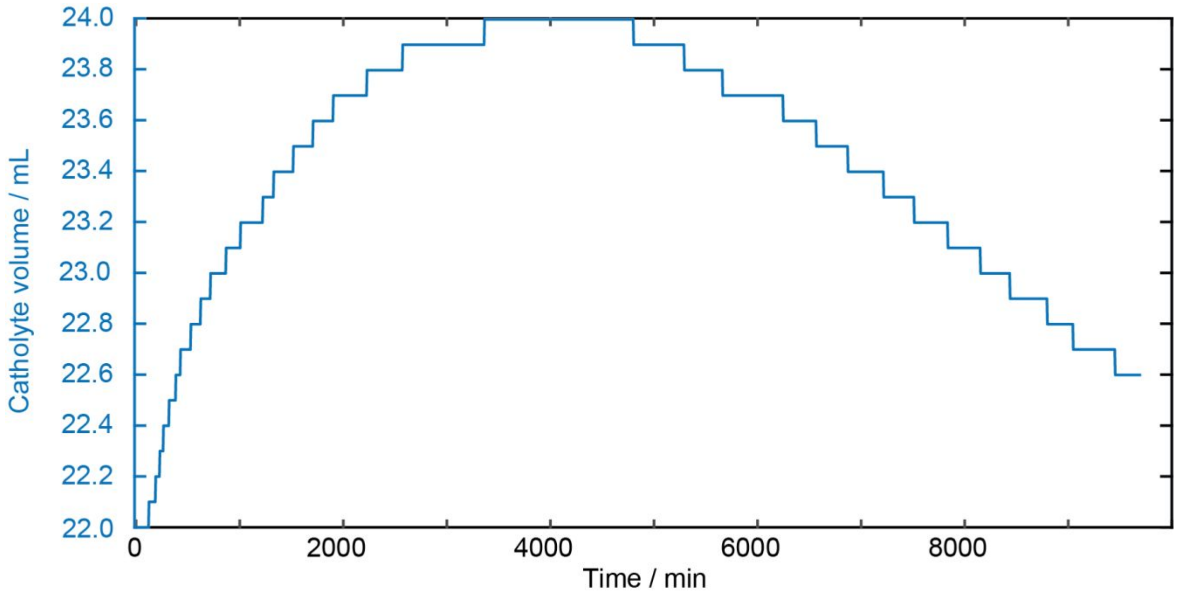

Figure S18: Plot showing measured catholyte level changes during cell rest.

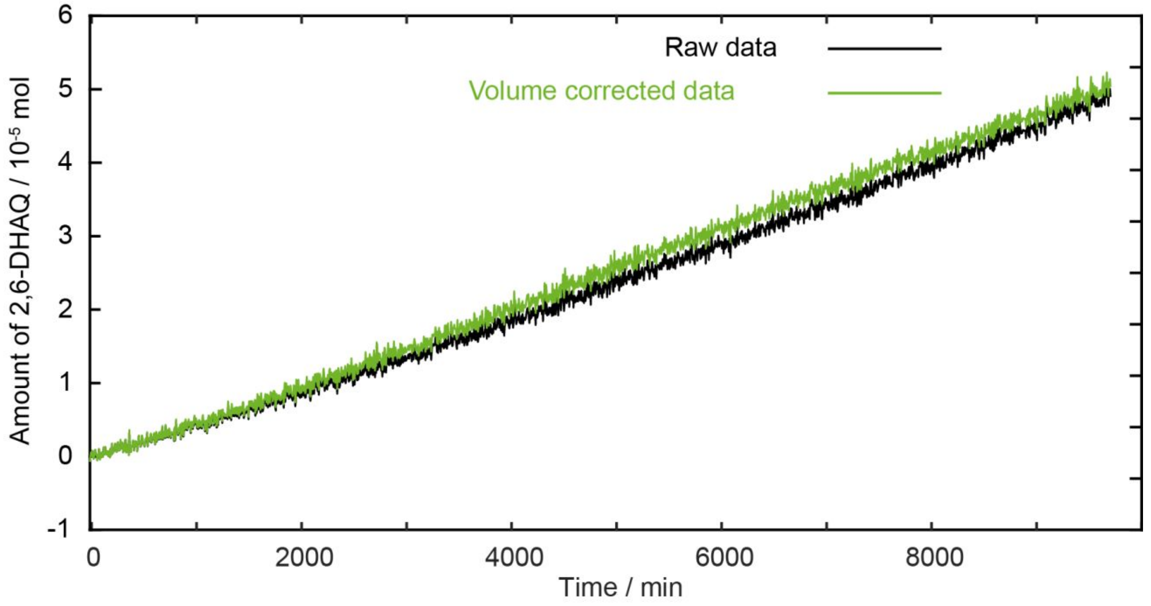

Figure S19: Impact of water crossover on 2,6-DHAQ crossover measurements at cell rest. The amount of 2,6-DHAQ crossover during cell rest is plotted in green and black for the volume corrected and raw data, respectively.

References:

(1) Zhao, E. W.; Liu, T.; Jónsson, E.; Lee, J.; Temprano, I.; Jethwa, R. B.; Wang, A.; Smith, H.; Carretero-González, J.; Song, Q.; Grey, C. P. In Situ NMR Metrology Reveals Reaction Mechanisms in Redox Flow Batteries. *Nature* **2020**, *579* (7798), 224–228. https://doi.org/10.1038/s41586-020-2081-7.

(2) Hall, A. M. R.; Chouler, J. C.; Codina, A.; Gierth, P. T.; Lowe, J. P.; Hintermair, U. Practical Aspects of Real-Time Reaction Monitoring Using Multi-Nuclear High Resolution FlowNMR Spectroscopy. *Catal. Sci. Technol.* **2016**, *6* (24), 8406–8417. https://doi.org/10.1039/C6CY01754A.

(3) Lin, K.; Chen, Q.; Gerhardt, M. R.; Tong, L.; Kim, S. B.; Eisenach, L.; Valle, A. W.; Hardee, D.; Gordon, R. G.; Aziz, M. J.; Marshak, M. P. Alkaline Quinone Flow Battery. *Science* **2015**, *349* (6255), 1529–1532. https://doi.org/10.1126/science.aab3033.

(4) Zhao, E. W.; Shellard, E. J. K.; Klusener, P. A. A.; Grey, C. P. *In Situ* Bulk Magnetization Measurements Reveal the State of Charge of Redox Flow Batteries. *Chem. Commun.* **2022**, *58* (9), 1342–1345. https://doi.org/10.1039/D1CC01895G.

(5) Evans, D. F. 400. The Determination of the Paramagnetic Susceptibility of Substances in Solution by Nuclear Magnetic Resonance. *J. Chem. Soc. Resumed* **1959**, 2003. https://doi.org/10.1039/jr9590002003.

(6) Arellano, C. A. P.; Martínez, S. S. Effects of pH on the Degradation of Aqueous Ferricyanide by Photolysis and Photocatalysis under Solar Radiation. *Sol. Energy Mater. Sol. Cells* **2010**, *94* (2), 327–332. https://doi.org/10.1016/j.solmat.2009.10.008.

(7) Hu, M.; Wang, A. P.; Luo, J.; Wei, Q.; Liu, T. L. Cycling Performance and Mechanistic Insights of Ferricyanide Electrolytes in Alkaline Redox Flow Batteries. *Adv. Energy Mater.* **2023**, *13* (15), 2203762. https://doi.org/10.1002/aenm.202203762.

(8) Fell, E. M.; De Porcellinis, D.; Jing, Y.; Gutierrez-Venegas, V.; George, T. Y.; Gordon, R. G.; Granados-Focil, S.; Aziz, M. J. Long-Term Stability of Ferri-/Ferrocyanide as an Electroactive Component for Redox Flow Battery Applications: On the Origin of Apparent Capacity Fade. *J. Electrochem. Soc.* **2023**, *170* (7), 070525. https://doi.org/10.1149/1945-7111/ace936.

(9) Zhao, E. W.; Liu, T.; Jónsson, E.; Lee, J.; Temprano, I.; Jethwa, R. B.; Wang, A.; Smith, H.; Carretero-González, J.; Song, Q.; Grey, C. P. In Situ NMR Metrology Reveals Reaction Mechanisms in Redox Flow Batteries. *Nature* **2020**, *579* (7798), 224–228. https://doi.org/10.1038/s41586-020-2081-7.

(10) Wu, B.; L. E. G. Aspers, R.; P. M. Kentgens, A.; Wenbo Zhao, E. Operando Benchtop NMR Reveals Reaction Intermediates and Crossover in Redox Flow Batteries. *J. Magn. Reson.* **2023**, 107448. https://doi.org/10.1016/j.jmr.2023.107448.

(11) Chu, K.-. C.; Xu, Y.; Balschi, J. A.; Springer, C. S. Bulk Magnetic Susceptibility Shifts in Nmr Studies of Compartmentalized Samples: Use of Paramagnetic Reagents. *Magn. Reson. Med.* **1990**, *13* (2), 239–262. https://doi.org/10.1002/mrm.1910130207.
