# Supplementary material for: Charge-Dependent Crossover in Aqueous Organic Redox Flow Batteries Revealed Using Online NMR Spectroscopy
Source: J Phys Chem Lett. 2024 Feb 1;15(5):1515–20. doi: 10.1021/acs.jpclett.3c03482 (PMC10860123; doi:10.1021/acs.jpclett.3c03482)
Supplement: Supplementary file 2 — jz3c03482_si_002.pdf [file jz3c03482_si_002.pdf]

Reviewer 1 Comments:

The manuscript "Charge-Dependent Crossover in Aqueous Organic Redox Flow Batteries Revealed Using On-Line NMR Spectroscopy" by Forse et al. proposed a new strategy to fullfill online NMR observation in AORFBs. The methods of sample preparation and signal quantification are reliable. The only comment I want to make is that fluid transportation from the catholyte tank to the NMR sample vial via a long transfer line can be really complex, especiallywhen the sample is pumped in and pushed out by fresher liquid. It would be ideal if the authors can comment on the effect of fluid mechanics and possible sample mixing within the transfer line and sample vial.

We fully agree that it is important to consider fluid dynamics in flow NMR studies, especially for quantification purposes. As we are looking for changes over longer time periods than the expected residence times within the tube, we think it is unlikely to significantly influence the results here. Fast response times are also observed in the changes in the battery state-of-charge (determined from the 1H chemical shift of the HOD peak) which correlate well with the battery charging protocol (Supporting Information Figure S6). Further discussion and a reference to a previous study (A. M. R. Hall et al.)1 on the fluid mechanics in the NMR flow tube has been added to page 6 of the Supporting Information, Methods S5:

"It is important to note that the catholyte residence time within the NMR flow tube is complicated by fluid dynamics. Under these conditions, it is likely that there will be laminar flow of the catholyte and some degree of back-mixing, which will increase the average residence time.[1] A. M. R. Hall et al.[1] observed non-ideal plug flow in the tip end of the InsightMR tube, which leads to a wider distribution in fluid residence times. This is one of the reasons it is important to use experimental methods to determine our quantitative flowrate and NMR parameters (Tables S4, Figure S4 and Figure S6), to ensure that impact of variable sample residence times and changes in magnetic field experienced by the sample before reaching the detection region were accounted for. The non-ideal flow in the NMR tube is unlikely to have a significant influence when measuring slow processes such as crossover, as we are observing changes over tens of minutes to hours. However, this flow behaviour could cause noticeable delays in the response times when measuring processes that occur on the order of seconds to minutes."

Reviewer 2 Comments:

This work demonstrates a new design of operando NMR strategy to investigate flow batteries under charging conditions. It shows the crossover from anolyte to catholyte is dependent on the charging current and the state of charge. The manuscript is well-writen and the results are solid. A suggestion to the authors is that the measurement may also be performed on the anolyte side. There could be some other interesting phenomena waiting to be uncovered.

It would certainly be interesting to apply these experiments to the anolyte side too. There are a number of detailed in situ NMR studies on reaction mechanisms and degradation of quinone electrolytes, which are referred to in the main text.[2–5] In regard to crossover, we have plans to use our setup to measure quinone size and charge changes as a function of battery state-of-charge, and how this could influence crossover rates. However, this requires some additional experiments using electrophoretic NMR which is beyond the scope of this proof-of-concept work. Where we have mentioned quinone effective size and charge, we have now added another sentence to suggest analysis on the anolyte side as future work. Main Text, page 10-11:

"This hypothesis could be tested further by performing diffusion-ordered NMR spectroscopy (DOSY) and electrophoretic NMR spectroscopy to the anolyte side of the battery when held at the same state-of-charge."[6,7]

It would also be interesting to track catholyte crossover into the anolyte, though as we are using a ferrocyanide catholyte here, this would be challenging. Ferricyanide does not contain any ^{1}H nuclei, and the available NMR active nuclei (^{13}C, ^{57}Fe, ^{14}N and ^{15}N) are unfavourable due to low sensitivity amongst other problems. It would also be difficult to track ferricyanide concentrations in the anolyte side based on HOD chemical shift, as a semiquinone radical anion is formed in the anolyte during charging, and this

would also influence chemical shift.[2] However, the AORFB field is generally moving to develop more organic catholytes to replace ferrocyanide and realise all-organic AORFBs. We have also added a sentence on this additional application of the method in the Main Text, page 11-12:

"Though we have focused on anolyte crossover here, this method is equally applicable to studying the crossover of novel organic catholytes."

References:

(1) Hall, A. M. R.; Chouler, J. C.; Codina, A.; Gierth, P. T.; Lowe, J. P.; Hintermair, U. Practical Aspects of Real-Time Reaction Monitoring Using Multi-Nuclear High Resolution FlowNMR Spectroscopy. Catal. Sci. Technol. 2016, 6 (24), 8406–8417. https://doi.org/10.1039/C6CY01754A.

(2) Zhao, E. W.; Liu, T.; Jónsson, E.; Lee, J.; Temprano, I.; Jethwa, R. B.; Wang, A.; Smith, H.; Carretero-González, J.; Song, Q.; Grey, C. P. In Situ NMR Metrology Reveals Reaction Mechanisms in Redox Flow Batteries. Nature 2020, 579 (7798), 224–228. https://doi.org/10.1038/s41586-020-2081-7.

(3) Zhao, E. W.; Jónsson, E.; Jethwa, R. B.; Hey, D.; Lyu, D.; Brookfield, A.; Klusener, P. A. A.; Collison, D.; Grey, C. P. Coupled In Situ NMR and EPR Studies Reveal the Electron Transfer Rate and Electrolyte Decomposition in Redox Flow Batteries. J. Am. Chem. Soc. 2021, 143 (4), 1885–1895. https://doi.org/10.1021/jacs.0c10650.

(4) Jing, Y.; Zhao, E. W.; Goulet, M.-A.; Bahari, M.; Fell, E. M.; Jin, S.; Davoodi, A.; Jónsson, E.; Wu, M.; Grey, C. P.; Gordon, R. G.; Aziz, M. J. In Situ Electrochemical Recomposition of Decomposed Redox-Active Species in Aqueous Organic Flow Batteries. Nat. Chem. 2022, 14 (10), 1103–1109. https://doi.org/10.1038/s41557-022-00967-4.

(5) Wu, B.; L. E. G. Aspers, R.; P. M. Kentgens, A.; Wenbo Zhao, E. Operando Benchtop NMR Reveals Reaction Intermediates and Crossover in Redox Flow Batteries. Journal of Magnetic Resonance 2023, 107448. https://doi.org/10.1016/j.jmr.2023.107448.

(6) Giesecke, M.; Mériguet, G.; Hallberg, F.; Fang, Y.; Stilbs, P.; Furó, I. Ion Association in Aqueous and Non-Aqueous Solutions Probed by Diffusion and Electrophoretic NMR. Phys. Chem. Chem. Phys. 2015, 17 (5), 3402–3408. https://doi.org/10.1039/C4CP04446K.

(7) Ackermann, F.; Schönhoff, M. Chelating Additives Reversing the Lithium Migration Direction in Ionic Liquid Electrolytes. J. Phys. Chem. C 2021, 125 (1), 266–274. https://doi.org/10.1021/acs.jpcc.0c09828.
